# The latest process and challenges of microwave dielectric ceramics based on pseudo phase diagrams

**DOI:** 10.1007/s40145-021-0528-4

**Published:** 2021-10-11

**Authors:** Hongcheng Yang, Shuren Zhang, Hongyu Yang, Qingyu Wen, Qiu Yang, Ling Gui, Qian Zhao, Enzhu Li

**Affiliations:** 1 National Engineering Research Center of Electromagnetic Radiation Control Materials, University of Electronic Science and Technology of China, Chengdu, 610054 China; 2 Key Laboratory of Multi-Spectral Absorbing Materials and Structures of Ministry of Education, University of Electronic Science and Technology of China, Chengdu, 610054 China

**Keywords:** high-temperature microwave dielectric ceramics (MWDCs), pseudo phase diagram, developments and challenges, composition-structure-property relationship

## Abstract

The explosive process of 5G communication evokes the urgent demand of miniaturized and integrated dielectric ceramics filter. It is a pressing need to advance the development of dielectric ceramics utilization of emerging technology to design new materials and understand the polarization mechanism. This review provides the summary of the study of microwave dielectric ceramics (MWDCs) sintered higher than 1000 from 2010 up to now, °C with the purpose of taking a broad and historical view of these ceramics and illustrating research directions. To date, researchers endeavor to explain the structure-property relationship of ceramics with multitude of approaches and design a new formula or strategy to obtain excellent microwave dielectric properties. There are variety of factors that impact the permittivity, dielectric loss, and temperature stability of dielectric materials, covering intrinsic and extrinsic factors. Many of these factors are often intertwined, which can complicate new dielectric material discovery and the mechanism investigation. Because of the various ceramics systems, pseudo phase diagram was used to classify the dielectric materials based on the composition. In this review, the ceramics were firstly divided into ternary systems, and then brief description of the experimental probes and complementary theoretical methods that have been used to discern the intrinsic polarization mechanisms and the origin of intrinsic loss was mentioned. Finally, some perspectives on the future outlook for high-temperature MWDCs were offered based on the synthesis method, characterization techniques, and significant theory developments.

## 1 Introduction

Over the past half century, semiconductor integration technology has become one of the most far-reaching and significant technological innovations in human society. The rapid development of this technology has enabled mankind to enter today's information society. However, semiconductor devices as active devices are only part of the electronic components. Another part of the huge amount and a wide variety of components with different functions are passive devices. The core materials of these components are various types of functional ceramic materials. Microwave dielectric ceramics (MWDCs) are the pivotal component of a passive device, which are mainly used as filters, resonators, RF antennae, frequency discriminators in electronic countermeasures, navigation, radar, home satellite live television receivers, and hand-held mobile phones. The applications of MWDCs in different frequency are directly plotted in Fig. 1. However, the development of microwave ceramics had gone through a sluggish procession because of the lack of suitable materials for dielectric resonator. The discovery of rutile (also known as titanium dioxide ceramics) in the 1970s makes it possible to synthesis dielectric resonator [1]. Various literature has been reported to explore the potential candidates of MWDCs after that, from single oxide, binary oxide, to ternary oxide. According to the data in the Web of Science, over 1000 papers were published about MWDCs around the world after 2000. Figure 2 presents the trend of published papers where more than 30% of investigations belong to China.

To evaluate the dielectric properties of ceramics, the relative permittivity ($\varepsilon_r$), dielectric loss (loss tangent or quality factor ($Q \times f$ value)), and temperature coefficient of resonant frequency ($\tau_f$) are the three pivotal characteristics. As early as in 2006, the direction of development of microwave dielectric materials has been highlighted by Ohsato *et al.* [2], including high $Q$ and low $\varepsilon_r$ ceramics for millimeter-wave application, high $Q$ and high $\varepsilon_r$ ceramics for base station, and high $\varepsilon_r$ ceramics for miniaturization of mobile phone. Up to now, researchers have explored hundreds of ceramics to enrich the database of MWDCs, but only a dozen of those ceramics with unique properties have been commercially used to fabricate relevant devices because most of the ceramics lack stability or generate large loss in the electronic components. Booming development of millimeter technology and 5G communication have rendered a new round of requirement of MWDCs of low permittivity with a stable dielectric loss in the scope

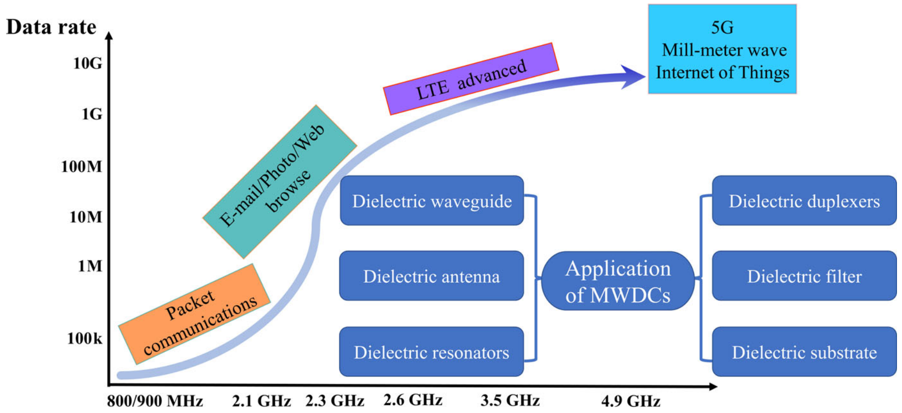

**Fig. 1** Applications of MWDCs in different frequencies.

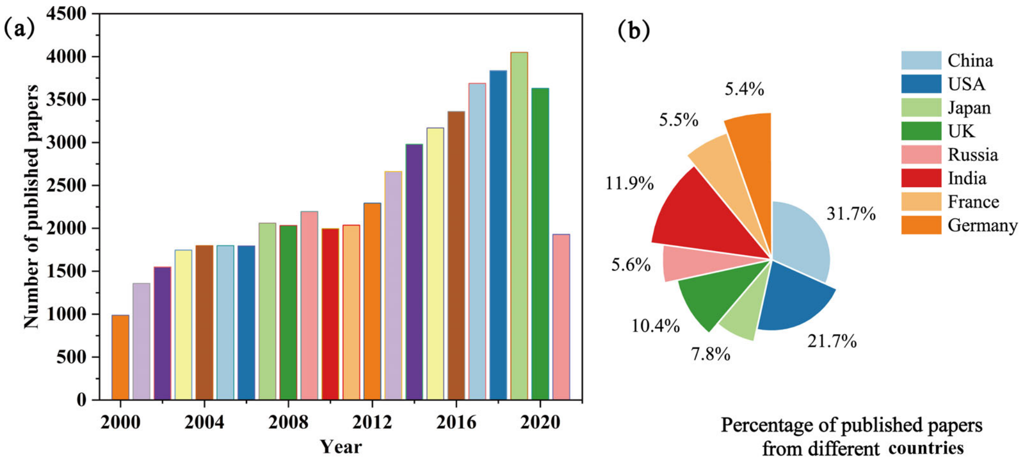

**Fig. 2** (a) Number of published papers about MWDCs from 2000 and (b) percentage of published papers from different countries.

of frequency up to 100 GHz. Especially, the emergency of COVID-19 makes video conferencing and telecommuting as a daily part in our lives. Consequently, the unprecedented growth of global data volume and huge demand for high data rates urge researchers to search more alternative materials for commercial electronic market. It is also a very significant issue for the industry to yield ceramics with ultra-low permittivity which are suitable for 5G and 6G communication system. However, it is still a "try and error" state in our experiments for discovering materials or optimizing the properties of the reported ceramics. The main difficulty in the development of MWDCs is to understand the fundamental relationship of composition–structure–property and draw general trends throughout the field, after normalizing and comparing the various results. Despite long-term sustained attempts, there is no systematic or comprehensive theory which can provide common guidance in the experiments and drive currently reported ceramics toward commercialization applications.

With the exploration of MWDCs clusters and the development of modern experiment techniques, investigations about MWDCs have been largely scoped by the designs and search for new systems and reoptimizing their properties. It is paramount that an MWDCs candidate has an appropriate dielectric constant, low dielectric loss, and near-zero temperature coefficient of resonant frequency for applications. Generally, to tune the microwave dielectric properties, there are two parts that should be taken into consideration (extrinsic and intrinsic parts). Extrinsic part is usually regarded as the influence originated from the synthesis method and raw materials. MWDCs usually prepare by solid state reaction method, and the sintering conditions directly influence the microstructure and compactness of ceramics, which subsequently affect the microwave dielectric properties. Meanwhile, the selectivity of size distribution, purity, non-stoichiometric ratio, species of different compounds, and pretreatment of raw materials based on their physical and chemical properties are crucial for reaching optimal microwave dielectric properties. For example, the procedures to reduce pores are designed for ceramics containing the volatile element, evolving non-stoichiometric ratio in the chemical formula, and providing the compensation atmosphere of volatile element. The relevant attempts are mostly discussed for the rock salt structure ceramics such as $Li_2Mg_3TiO_6$. Besides, various synthesis methods, namely sol–gel method, sink plasma sintering method, and high energy ball-milling method are gradually used for preparing the MWDCs, and numbers of studies analyze the discrepancy of microwave dielectric properties obtained with different methods. The intrinsic part stems from anharmonic lattice vibration, which primarily generates large dielectric loss. As yet, there is no technology or theory that could feasibly adjust the anharmonic lattice vibration to reduce dielectric loss. In the experiment, after carefully controlling the sintering conditions and selecting raw materials, the most pragmatic approach to optimize the properties is cation substitution with the consideration of the radii and the electronegativity of cations, contributing to reducing the dielectric loss or modifying the temperature coefficient of resonant frequency. Near-zero temperature coefficient of resonant frequency is also obtained by designing co-exited phase system with introduction of two ceramics with opposite $\tau_f$ values, but the composite ceramics may lead to a poor $Q\times f$ value. More recently, the strategy of tri-layer structures of $Zn_{1.01}Nb_2O_6/TiO_2/Zn_{1.01}Nb_2O_6$ [3], $MgTiO_3/TiO_2/MgTiO_3$ [4], and $Zn_3Nb_2O_8/TiO_2/Zn_3Nb_2O_8$ [5] were verified as a method to obtain the temperature-stable ceramics with low dielectric loss.

Currently, the database of MWDCs is enriched by insightful information about the structure and properties, and the growing number of literature converts from description of phenomena to explanation of the theoretical mechanism of the dielectric materials. Thorough and comprehensive investigation of ceramics is gradually presented to estimate the extrinsic and intrinsic influence on the microwave dielectric properties. For instance, the common discussion of polarization mechanism is usually based on the ionic polarization, where the Clausius–Mossotti (C–M) equation is applied to evaluate the discrepancy of theoretical $\varepsilon_r$ and measured $\varepsilon_r$. The popularization of Rietveld-refinement in the literature supports the analysis of lattice parameters, packing fraction, and chemical bond characteristic obtained by the complex chemical bond theory (P–V–L) theory. Especially, disassembling the crystal into the sum of sample binary compound based on the crystal parameters and coordinate numbers of each ions [6], the investigations about application of P–V–L theory into multi-type structure emerge in abundance. The origin of dielectric loss is quantified by lattice vibrational spectroscopy, and the contribution of each chemical bond to the microwave dielectric properties is verified by P–V–L theory. For some unique ceramics, researchers bend themselves to exploring the underlying mechanism for the observed phenomenon. The influence of long-range movement of charged defects in the grain and grain boundary was estimated by the impedance analysis, terahertz (THz) time-domain spectroscopy analysis, and the electron paramagnetic resonance spectra, which can explain the defect generation mechanism in doped $Li_2ZnTi_3O_8$ ceramics. The analysis of disordered–ordered crystal structure evolution and super-lattice in rock salt ceramics and complex perovskite ceramics gives evidence to explain the ultra-low dielectric loss. Both the development of experimental and theoretical method allows us to summarize the relevant experimental probes of different systems and propose the challenges and prospects of MWDCs.

While many great review and perspective articles have been published about MWDCs, they have finished the review by classified MWDCs based on the criteria of sintering temperature, dielectric constant, and crystal structure [1,7–9]. Furthermore, the early works before 2010 are mainly concentrated on the description of phase composition, micrographic images, and variation of microwave dielectric properties. The topic about the MWDCs sintered lower than 950 ℃ is especially focused owing to the advantages of low-temperature co-fired ceramics (LTCC) technology where this approach guarantees the integration of electronic components. Considering either the timespan or topic covered, the mentioned ceramics, in this review, are all sintered higher than 1000 ℃. The LTCC system including ceramics with a few sintering aids, glass–ceramics system, or glass-free system is not referred. To follow the development of new analysis methods, MWDCs, beginning with the first reported properties and upgrading the relevant references after 2010, were included. Additionally, because of so various structures and properties of MWDCs, pseudo phase diagram was used to classify the ceramics according to the composition, which will serve as the basis and link for each pseudo phase diagram of diversity composition. The organization of this review consists of a brief section detailing the phase evolution or structure transformation of oxide ceramics in the designed pseudo phase diagram, and then the chronological experimental probes for a unique system are summarized.

## 2 Phase diagram

The phase diagram is a visual representation of the phase equilibrium, which defines the composition of multiphase system. It is an efficient and convenient technique to analyze the composition and their proportion, which plays a significant role in guiding the research and exploration of materials to reduce the manpower and material resources effectively. This section provides a broad context by summarizing the ceramics system based on pseudo phase diagram, and all the composition in the following pseudo phase diagram is in molar ratio. The endpoint of each pseudo phase diagram contains more than one component, and the labelled ceramics are the primary system reported by researchers. The summary of investigations in the same general formal is listed in detail.

### 2. 1 Silicate and germanate

There is a low $\varepsilon_r$ (< 10) for silicates, owing to the low ionic polarizability of $Si^{4+}$ and half covalent bond in Si–O. In the binary silicate, the $CaSiO_3$, $Mg_2SiO_4$, $Zn_2SiO_4$, and $Re_2SiO_5$ are the main representatives, where $CaSiO_3$ usually appeared as the crystalized phase in $CaO–B_2O_3–SiO_2$ glass. Ternary silicate such as diopside-type $CaMgSi_2O_6$, melitile-type $A_2BC_2O_7$ and $AB_2C_2O_7$ (A = Sr, Ca, Ba; B = Mg, Zn, Co, Mn, Cu), and cuspidine-type $Ca_3SnSi_2O_9$ were highlighted by researchers, due to the diverse crystal structures in those systems. With the wake of exploration of new ceramics, the germanate gradually occurred as a candidate material with low dielectric loss despite of the expensive cost of $GeO_2$ as raw material. The pseudo phase diagram of the silicate and germanate is presented in Fig. 3, where the primary phases of binary and ternary silicate and germanate are listed in the phase diagram.

#### *2.1.1 Binary silicate ceramics*

Synthesis of dense $SiO_2$ ceramic is challengeable because of its complexity in polymorphs and phase transitions. Until 2012, microwave dielectric properties of $SiO_2$ ceramic were reported as $\varepsilon_r \approx 3.81$, $Q\times f$ value ≈ 80,400 GHz, and $\tau_f \approx -16.1$ ppm/℃, sintered at 1650 ℃ for 3 h [10]. After that, Li *et al*. [11] illustrated that $0.84SiO_2–0.16TiO_2$ composite ceramics possessed satisfied properties of $\varepsilon_r \approx 5.91$, $Q\times f$ value ≈ 39,680 GHz, and $\tau_f \approx -4.5$ ppm/℃, sintered at 1275 ℃ for 3 h.

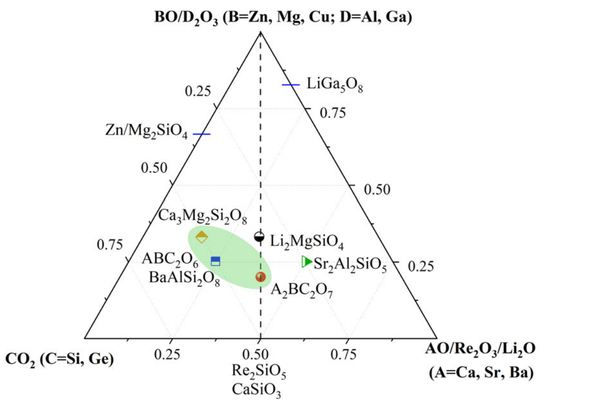

**Fig. 3** Pseudo phase diagram of the silicate and germinate.

Comparing to the difficulty of preparing compact $SiO_2$ ceramic, the restriction of preparing dense $CaSiO_3$ ceramic stemmed from the narrow sintering temperature range of pure $CaSiO_3$ and the porous microstructure [12]. Commonly, $CaSiO_3$ was reported as a main phase in the $CaO–B_2O_3–SiO_2$ glass–ceramic system, which primarily determined the properties. There are two main phases of $CaSiO_3$, containing low-temperature wollastonite ($\alpha$-$CaSiO_3$) and high-temperature preudo-wollastonite ($\beta$-$CaSiO_3$). Through a sol–gel method, the microwave dielectric properties of $\alpha$-$CaSiO_3$ are: $\varepsilon_r \approx 6.69$, $Q\times f$ value ≈ 25,398 GHz, sintered at 1320 ℃ [13]. In order to improve the microwave dielectric properties, $(Ca_{1-x}Mg_x)SiO_3$ ceramics with $x = 0.1$, 0.5, 0.9 were verified as single phases, and properties of $\varepsilon_r \approx 6.49$, $Q\times f$ value ≈ 62,420 GHz, and $\tau_f \approx -43.3$ ppm/℃ were obtained when sintered at 1320 ℃ with $x = 0.1$ [14]. Besides, the investigation of $CaSiO_3–Al_2O_3$ ceramics revealed that the secondary phases of $Ca_2Al_2SiO_7$ and $CaAl_2Si_2O_8$ would deteriorate the microwave dielectric properties with excessive $Al_2O_3$ [15,16]. According to Hu *et al.* [17], the phase transformation of $CaSiO_3$ was inhibited with the increase of $SiO_2$ content, and $\alpha$-$CaSiO_3$–2 wt% $Al_2O_3$–2.5 wt% $TiO_2$ shows excellent properties of $\varepsilon_r \approx 7.88$, $Q\times f$ value ≈ 24,412 GHz, and $\tau_f \approx -0.52$ ppm/℃ [18]. To obtain compact ceramics, $SnO_2$-doped $\alpha$-$CaSiO_3$ ceramics with $\varepsilon_r \approx 9.27$, $Q\times f$ value ≈ 53,000 GHz, and $\tau_f \approx -52$ ppm/℃ sintered at 1450 ℃ were reported in a relative density higher than 97% [19].

With a $Q\times f$ value larger than 100,000 GHz, another class of binary silicate can be written as $A_2SiO_4$ (A = Ba, Sr, Ca, Mg, Zn) [20–23]. Forsterite $Mg_2SiO_4$ is extensively explored because of its superior microwave dielectric properties ($\varepsilon_r \approx 7.8$, $Q\times f$ value ≈ 240,000 GHz, and $\tau_f \approx -67$ ppm/℃, sintered at 1450–1500 ℃) [2,24,25]. Nevertheless, the high sintering temperature and large $\tau_f$ value inhibit its application. To adjust $\tau_f$ value, both co-exited phase of $Mg_2SiO_4–Ca_{0.9}Sr_{0.1}TiO_3$ [26] and $Zn_2SiO_4–TiO_2$ [27] contributed to a near-zero $\tau_f$ value. Melting CuO could enhance the sintering procession of $Zn_2SiO_4$, and the quality factor reached 105,500 GHz when sintered at 1150 ℃ [28]. Zn-deficient formula was verified valid to suppress the formation of secondary phase in $Zn_2SiO_4$ ceramics, and $Zn_{1.8}SiO_{3.8}$ was estimated with properties of $\varepsilon_r \approx 6.451$, $Q\times f$ value ≈ 102,807 GHz, and $\tau_f \approx -32$ ppm/℃, sintered at 1300 ℃ [29].

After predicting the permittivity of $ZrO_2–SiO_2$, $HfO_2–SiO_2$, $La_2O_2–SiO_2$, and $Y_2O_3–SiO_2$, those systems were clarified as an alternative of dynamic random access memory capacitor dielectric materials [30]. The exploration of properties of $Sm_2SiO_5$ and $Nd_2SiO_5$ ceramics compensated the absence of study on microwave dielectric properties of $Re_2O_3–SiO_2$, where the microwave dielectric properties were listed as $\varepsilon_r \approx 8.44$, $Q\times f$ value ≈ 64,000 GHz, and $\tau_f \approx -37$ ppm/℃ and $\varepsilon_r \approx 7.94$, $Q\times f$ value ≈ 38,800 GHz, and $\tau_f \approx -53$ ppm/℃ with the molar ratio of $Re_2O_3/SiO_2$ = 1:1.05, respectively [31,32].

### *2.1.2 Ternary silicate and germanate ceramics*

Clinopyroxene-type $ABC_2O_6$ (A = Ca; B = Co, Mg, Zn, Fe; C = Si, Ge) materials, akermanite-type $A_2BC_2O_7$ (A = Sr, Ca; B = Mg, Zn, Co, Mn; C = Si, Ge), and melilite-type $A_2BSi_2O_7$ (A = Sr, Ca, Ba; B = Mg, Zn, Co, Mn, Cu) occupied the primary family of ternary silicate ceramics. Increasing attention has been paid for $CaMgSi_2O_6$ owing to its low permittivity ≈ 7.5, which is suitable to be substrate [14,33–35]. Both the substitution of $Zn^{2+}$, $Co^{2+}$, $Cu^{2+}$, $Mn^{2+}$ for $Mg^{2+}$ and introduction of $Sr^{2+}$ into $Ca^{2+}$ of $CaMgSi_2O_6$ were benefit for reducing the dielectric loss. Microstructure with many pores of $CaMnSi_2O_6$ was observed by Chen *et al.* [36], and the effect of porosity on the properties was investigated by spherical-pore model. Akermanite-type $A_2BC_2O_7$ (A = Sr, Ca; B = Mg, Zn, Co, Mn; C = Si, Ge) systems belong to the structure group of $P\bar{4}2_1m$ (113) in tetragonal, while melilite-type $A_2BC_2O_7$ (A = Ba; B = Co, Zn, Cu, Mg; C = Si, Ge) and $AB_2C_2O_7$ (A = Ba; B = Co, Zn; C = Si, Ge) systems were clarified in monoclinic structure [37–44]. The literature about the effect of structure evolution and chemical bond parameters in $A_2BSi_2O_7$ and $AB_2Si_2O_7$ represented that the Si–O bond played the significant role in structural stability and dielectric polarization.

The monoclinic $Ca_3SnSi_2O_9$ and $Ca_3MgSi_2O_8$ with space group $P2_1/c$ were investigated to supplement the compound of ternary silicate oxides. $Ca_3SnSi_2O_9$ ceramics were obtained in a wide sintering temperature region from 1400 to 1525 ℃, with non-stoichiometric composition (molar ratio of Ca:Sn:Si = 1:1.2:1) as raw materials [45]. Single phase $Ca_3MgSi_2O_8$ possessed near 99% of the theoretical density after sintered at 1375 ℃, with $\varepsilon_r \approx 13.8$, $Q\times f$ value ≈ 27,000 GHz, and $\tau_f \approx -62$ ppm/℃ [46]. Sintering behavior and phase composition of gillespite-structured $MCuSi_4O_{10}$ (M = $Ba_{1-x}Sr_x$, $Sr_{1-x}Ca_x$) ceramics were established by Song *et al.* [47], and $SrCuSi_4O_{10}$ possessed microwave dielectric properties of $\varepsilon_r \approx 5.59$, $Q\times f$ value ≈ 82,252 GHz, and $\tau_f \approx -41.34$ ppm/℃. The first-principles calculation was applied to determine where $Ni^{2+}$ and $Li^+$ would occupy in $BaAl_2Si_2O_8$ ceramics, and the change of bond strength and bond valence were analyzed [48,49].

The unpresented ternary silicate and germanate phase in pseudo phase diagrams are summarized as well in this section. The rare earth-based ternary silicate oxides, such as apatite with general formula $A_{10}(MO_4)_6O_2$ (A = alkaline earth, rare earth, Pb; M = Si, Ge, P, V), have received much attention since the apatite structure allowed numbers of substitutions at all the three sites. The lattice parameters and the local charge compensation of apatite type compounds were determined in 1972 [50], and those ceramics were established as candidate of fluorescent lamp phosphors and laser technology. To improve the densification of lithium apatite $LiRe_9(SiO_4)_6O_2$ ceramics (Re = La, Pr, Nd, Sm, Eu, Gd, Er), relative density was higher than 90% for all samples after doping 1 wt% LiF [51]. The microwave dielectric properties of $SrRE_4Si_3O_{13}$ (RE = La, Pr, Nd, Sm, Eu, Gd, Tb, Dy, Er, Tm, Yb, and Y) were in the range of 9–16 for permittivity with the maximum of $Q\times f$ value ≈ 26,000 GHz [52], while the optimal microwave dielectric properties of $CaRE_4Si_3O_{13}$ (RE = La, Nd, Sm, and Er) were $\varepsilon_r \approx 13.37$, $Q\times f$ value ≈ 18,600 GHz, and $\tau_f \approx -17.8$ ppm/℃ at Re = Er [53].

To obtain new dielectric materials, some researchers pursued materials with the composition containing $GeO_2$ and $Ga_2O_3$ and reported microwave dielectric properties of those materials firstly. With inverse spinel structure, $LiGa_5O_8$ was verified as a cubic structure where $Li^+$ and $Ga^{3+}$ distributed in the octahedral B site with 1:3 ordering [54]. The large deviation between $\varepsilon_r$ and $\varepsilon_{rth}$ in $Ba_2MGa_{11}O_{20}$ (M = Bi, La) was ascribed to the "rattling" effect of cations and the existence of lone pair ions of $Bi^{3+}$ [55]. The different $\tau_f$ values of $AGe_4O_9$ (A = Ba, Sr) were ascribed to the distortion of $[GeO_6]$ octahedron where $\tau_f$ values were –44.2 ppm/℃ for the former and –11.7 ppm/℃ for the later [56]. Normal garnet $A_3Y_2Ge_3O_{12}$ (A = Ca, Mg) ceramics possessed $\tau_f \approx 120.5$ ppm/℃ for A = Ca and –40.6 ppm/℃ for A = Mg [57]. As doped ions, $(Li_{0.5}Ga_{0.5})^{3+}$ in $Mg_2Al_4Si_5O_{18}$ would obtain the highest $Q\times f$ value of 50,560 GHz [58]. $Ca_3M_2Si_3O_{12}$ (M = Yb, Y) ceramics were consistent with the general formula of garnet structure, and those ceramics crystalized as silico-carnotite structure with high-energy ball milling method [59]. The microwave dielectric properties were recorded as $\varepsilon_r \approx 9.2$, $Q\times f$ value ≈ 56,400 GHz, and $\tau_f \approx -77.5$ ppm/℃ and $\varepsilon_r \approx 8.7$, $Q\times f$ value ≈ 29,094 GHz, and $\tau_f \approx -76.8$ ppm/℃ for $Ca_3Yb_2Si_3O_{12}$ and $Ca_3Y_2Si_3O_{12}$, respectively. A serial of $Ca_3MZrGe_3O_{12}$ (M = Co, Zn), $Ca_4ZrGe_3O_{12}$, and $Ca_3B_2Ge_3O_{12}$ (B = Al, Ga) ceramics were successfully prepared, and the quality factors were higher than that of $Ca_3M_2Si_3O_{12}$ [60–62]. Similarly, $Sr_3B_2Ge_3O_{12}$ (B = Yb, Ho) were investigated by Li *et al.* [63] using vibration spectroscopy, and the $\tau_f$ was tuned to near zero with $CaTiO_3$ ceramics. $0.8Y_3MgAl_3SiO_{12}$–$0.2TiO_2$ ceramic sintered at 1475 ℃ showed a $\tau_f \approx +5.2$ ppm/℃, where the co-existed phase contained $Y_2Ti_2O_7$ and $TiO_2$ along with $Y_3MgAl_3SiO_{12}$ phase [64]. Dense $Mg_3Ga_2GeO_8$ ceramics presented microwave dielectric properties of $\varepsilon_r \approx 9.41$, $Q\times f$ value ≈ 133,113 GHz, and $\tau_f \approx -63.54$ ppm/℃ [65]. Single phase $LiYSiO_4$ ceramics could be obtained in 1100–1140 ℃, and a near-zero $\tau_f$ of (+4.52)–(+8.03) ppm/℃ was observed [66].

Furthermore, phase transition from $A2/a$ to $P2_1/a$ was observed in new silicate in the formula of $CaSn_{1-x}Ti_xSiO_5$, where the variation of $\tau_f$ values was ascribed to the Sn/$TiO_6$ octahedral distortion [67]. Secondary phase of $SnO_2$ and $SrSiO_3$ appeared at $0.2 \leqslant x \leqslant 0.45$ in $Ca_{1-x}Sr_xSnSiO_5$ ceramics, which could adjust the positive $\tau_f$ of $CaSnSiO_5$ to –1.2 ppm/℃ [68]. $CaSiO_3$ and $CaSnSiO_5$ phases would improve the $\tau_f$ to –7.2 ppm/℃ in $Ca_2(Hf_{1-x}Sn_x)Si_4O_{12}$ when $x = 0.4$ [69].

### 2.2 Niobate and tantalate based on $ZnO$–$Nb_2O_5$–$TiO_2$

There is a large body of niobate and tantalate dielectric ceramics, and the relevant researches highlight the phase evolution, structure transformation, and chemical bond characteristics. In order to elucidate the influence of undercoordinated sites on the dielectric properties, analysis according to P–V–L theory and vibration spectra is verified as valid approach to understand the relationship of the state of chemical bond with polarization and stability of lattice. Indeed, it seems that researchers could identify the contribution of each chemical bond to dielectric properties by P–V–L theory and infrared reflectivity spectrum. However, reaching general conclusions about the effect of a unique chemical bond or Wycoff site on different properties may be difficult, since the P–V–L theory is just predictable theoretically. The actual dielectric properties of ceramics are still evaluated based on experiments, and thorough, quantitative, and multi-perspective analysis is required. Figure 4 is the phase diagram of the mainly reported niobate and tantalate dielectric ceramics, where the rutile-type, ixiolite-type, and columbite-type structures were obtained after $(Zn_{1/3}Nb_{2/3})^{4+}$ was doped into $TiO_2$. The detailed phase division of $A_{0.5}B_{0.5}CO_4$ and the relevant investigations of this binary system are summarized in the following.

### *2.2.1 Rutile–trirutile/ixiolite/wolframite–columbite type ceramics*

Rutile, brookite, and anatase are the three types of $TiO_2$ in nature. $TiO_2$ itself possesses a high permittivity ≈ 100, a low dielectric loss tangent (tan$\sigma$) value ($6\times10^{-5}$ at a frequency of 3 GHz), and a high $\tau_f$ value of 450 ppm/℃ [70]. It is valid that $TiO_2$ phase is used to target the aim of near zero $\tau_f$ value as a secondary phase in the system with a negative $\tau_f$ value. Meanwhile, long-term focus has been paid on the structure transformation and property optimization of $TiO_2$ with

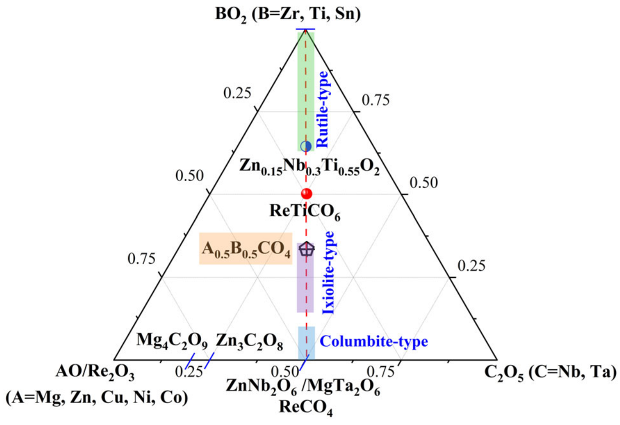

**Fig. 4** Pseudo phase diagram of the niobate and tantalite.

substitution ions of different physicochemical properties. The cation substitution for $Ti^{4+}$ can reduce the dielectric loss or tune the $\tau_f$ value, evolving monovalent, divalent, trivalent, tetravalent, or pentavalent cations, and their groups of two cations. Especially, the extensive elaboration of dependence of microwave dielectric properties on the crystal structure of $(Zn_{1/3}B^{5+}_{2/3})_xTi_{1-x}O_2$ ($B^{5+}$ = Nb, Ta) ceramics was reported by Kim and Kang [71]. The phase relation of ternary system of $ZnO–TiO_2–Nb_2O_5$ was first discussed in 1992 [72]. It summarized that the solid solution of rutile phase appeared in the range of molar content of $(Zn_{1/3}Nb_{2/3})^{4+}$ lower than 58%, ixiolite $ZnTiNb_2O_8$ exited in the range of 69%–85%, while columbite solid solution of $ZnNb_2O_6$ formed when the content was higher than 93% [73], and the solid solution area of different types was marked with shadow in the pseudo phase diagram in Fig. 4.

Compared with the ongoing report of $ZnTiNb_2O_8$, the study of $Zn_{0.15}Nb_{0.3}Ti_{0.55}O_2$ is still rare. Generally, $Zn_{0.15}Nb_{0.3}Ti_{0.55}O_2$ appeared as the secondary phase which would control the dielectric properties of composite ceramics [74–76]. It possessed properties of $\varepsilon_r$ ≈ 94.35, $Q\times f$ value ≈ 10,889 GHz, and $\tau_f$ ≈ 353.43 ppm/℃, sintered at 1050 ℃ [71], which was potential to be $\tau_f$ compensator as $TiO_2$. Yang *et al.* [77] directly added the $Zn_{0.15}Nb_{0.3}Ti_{0.55}O_2$ into $Zn_{0.5}Ti_{0.5}NbO_4$ ceramics, and the structure evolution and chemical bond parameters have been calculated. $Zr^{4+}$ with the larger radius than $Ti^{4+}$ was used to dope into $Zn_{0.15}Nb_{0.3}(Ti_{1-x}Zr_x)_{0.55}O_2$ [78], where the expansion of bond length and cell volume renders the decline of covalency of all bonds. The decline of bond ionicity was obtained since the shrinking of cell volume and bond length in $Zn_{0.15}Nb_{0.3-x}Ta_xTiZr_{0.55}O_2$ [79].

The structure of formula $A_{0.5}B_{0.5}CO_4$ can be categorized into four types: wolframite-type $AZrB_2O_8$ (A = Mn, Zn, Mg, Co, Ni; B = Nb, Ta), rutile-type $A_{0.5}Ti_{0.5}NbO_4$ (A = Ni, Co, Cu), tetragonal trirutile-type $A_{0.5}Ti/Sn_{0.5}TaO_4$ structure (A = Co, Ni, Zn, Mg), and ixiolite-type $ZnTiNb_2O_8$. The schematic of those classifications is shown in Fig. 5, and the related investigations of each structure are illustrated in this section. The effects of different cations (Mn, Zn, Mg, Ni, and Co) at A-site of $AZrNb_2O_8$ illustrated that dielectric constant, quality factor, and $\tau_f$ values relied on the ionic polarizability, packing fraction, and B-site octahedral distortions, respectively [80–85]. Among them, $MgZrNb_2O_8$ shows the optimal quality factor

($\varepsilon_r \approx 26$, $Q\times f$ value ≈ 120,816 GHz, and $\tau_f \approx -50.2$ ppm/℃, at $f$ = 6.85 GHz [86]). The microwave dielectric properties of wolframite-type $AZrB_2O_8$ (A = Mn, Zn, Mg, Co, Ni; B = Nb, Ta) and the structure-relationship were determined via combining the far-infrared and terahertz spectroscopy with P–V–L theory [87–91]. Partial replace of A-site (such as $Mg_{0.5}Zn_{0.5}ZrNb_2O_8$ [92], $Zn_{1-x}Co_xZrNb_2O_8$ [93–95]), Zr-site substitution of $Zn(Ti_{1-x}Zr_x)Ta_2O_8$ [96], $ZnZr_{1-x}Sn_xNb_2O_8$ [97,98], doped Nb-site of $MgZr(Nb_{1-x}Sb_x)_2O_8$ [99,100], $ZnZrNbTaO_8$ [101], $MgZrNb_{2-x}(Sn_{1/2}W_{1/2})_xO_8$ [102], and non-stoichiometric $MgZrNb_{2+x}O_{8+2.5x}$ [103] provided evidence that relative density, packing fraction, bond valence, and chemical bond characteristics majored the variation of microwave dielectric properties. To adjust the negative $\tau_f$ values, the study about the relationship of $TiO_2$ on $MgZrNb_2O_8$ [104] and $ZnZrNb_2O_8$ [105] presented that co-exited ceramics would reach near zero $\tau_f$ values. The microwave dielectric properties were $\varepsilon_r \approx 43$, $Q\times f$ value ≈ 46,110 GHz, and $\tau_f \approx -2.5$ ppm/℃ for $0.63MgZrNb_2O_8$–$0.37TiO_2$ ceramics; $\varepsilon_r \approx$ 44, $Q\times f$ value ≈ 38,500 GHz, and $\tau_f \approx -2.4$ ppm/℃ for $0.3ZnZrNb_2O_8$–$0.7TiO_2$ ceramics. Additionally, literature demonstrated that $H_3BO_3$ or $B_2O_3$ addictive aids could contributed to densification and improvement of the sintering behavior for $ZnZrNb_2O_8$ and $MgZrNb_2O_8$ [106–108].

The dielectric properties of $A_{0.5}B_{0.5}NbO_4$ ceramics are much different. $Ni_{0.5}Ti_{0.5}NbO_4$ and $Cu_{0.5}Ti_{0.5}NbO_4$ crystalized in rutile structure presented with positive $\tau_f$ values of 79.1 and 49.2 ppm/℃, respectively [109,110]. The characteristic of rutile $Co_{0.5}Ti_{0.5}NbO_4$ was sought by solid state reaction and sol–gel method [111,112], where the microwave dielectric properties were $\varepsilon_r \approx 64$, $Q\times f$ value ≈ 65,300 GHz, $\tau_f \approx 223.2$ ppm/℃ and $\varepsilon_r \approx$ 64.19, $Q\times f$ value ≈ 16,800 GHz, $\tau_f \approx 66.17$ ppm/℃, respectively. In the solid solution of $Ni_{0.5-x}Zn_xTi_{0.5}NbO_4$, the dielectric constant was enhanced from 56.8 to 62.54 [113]. Introduction of $CoNb_2O_6$ and $Zn_{1.01}Nb_2O_6$ into $CoTiNb_2O_8$ rendered the $Q\times f$ increasing considerably due to the enhanced densification and obtained the $\tau_f$ values of 0.5 and 0 ppm/℃, respectively [114,115]. Zhang *et al*. [116] and Li *et al*. [117] reported that $\tau_f$ value would shift from positive to negative after Zr substitution in $CoTi_{1-x}Zr_xNb_2O_8$, where the $\tau_f$ value was correlated with oxygen octahedral distortion and B-site bond valence. Superlattice diffraction peak which is relevant with cation ordering was observed in $Co_{0.5}Ti_{0.5}Nb_{1-x}Sb_xO_4$ ceramics, contributing to the augment of $Q\times f$ value [118].

The trirutile-type structure was observed in some tantalates, antimonates, and bismuthates. This crystal structure was built by ordering octahedral cations along *c*-axis, which possessed three times *c*-axis of rutile-type one [119,120]. Currently, $Co_{0.5}Ti_{0.5}TaO_4$ [121], $NiTiTa_2O_8$ [122], $Co_{0.5}Zr_{0.5}TaO_4$ [90], $NiSnTa_2O_8$ [123] were reported as trirutile-type structure. Among them, $NiSnTa_2O_8$ showed a near zero $\tau_f$ value ($\varepsilon_r \approx 21.04$, $Q\times f$ value ≈ 31,328 GHz, and $\tau_f \approx -2.63$ ppm/℃).

Ixiolite phase $ZnTiNb_2O_8$ is a fully disordered α-$PbO_2$ structure, where Zn/Ti/Nb ions statistically occupied the octahedral cation sites [124]. Up to now, numbers of substitution on $ZnTiNb_2O_8$ have been reported, such as Co [125], Mg [74], Ca [126], Sn [127], Zr [128], and Ta [129–131]. The crystal structure refinement and Raman spectrum study of $ZnTiNb_2O_8$, together with the mode assignment were completed by Liao and Li [132]. In the $ZnO$–$Nb_2O_5$–$xTiO_2$ ($1 \leqslant x \leqslant 2$) system, ceramics were composed of $Zn_{0.17}Nb_{0.33}Ti_{0.5}O_2$ and $ZnTiNb_2O_8$ when $x \geqslant 1.8$ [133]. Using the effective

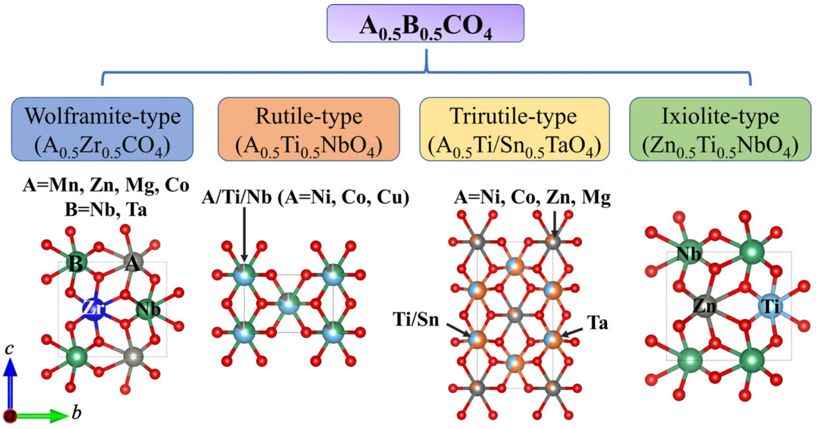

**Fig. 5** Schematic of classification of $A_{0.5}B_{0.5}CO_4$.

route of sintering reaction for $ZnNb_2O_6$ and $TiO_2$ nano powders, a superior property of $ZnTiNb_2O_8$ was achieved compared with that prepared by solid-state method [134]. Dielectric constant and dielectric loss were evaluated in microwave and THz range in $Al_{0.5}Nb_{0.5}$ doped into $ZnTiNb_2O_8$, where the results indicated the negligible shift of dielectric constant in those frequencies, as shown in Fig. 6 [135]. Furthermore, ixiolite $MgTiNb_2O_8$ prepared by aqueous sol–gel process and then sintered at 1000 ℃ showed $\varepsilon_r \approx 33.8$, $Q\times f$ value ≈ 26,260 GHz, and $\tau_f \approx -19.2$ ppm/℃ [136].

In the family of $AB_2O_6$ (A = Ca, Mg, Mn, Co, Ni, Zn; B = Ta, Nb), the relationship of permittivity with electronegativity was presented by Lee *et al.* [137]. Two structure classifications have been identified in this system, namely rutile-type (trirutile) and α-$PbO_2$-type (tri-α-$PbO_2$, columibite) [138,139]. Comprehensive studies of columbite niobates concluded that the $\varepsilon_r$ was in the range of 17–22, $\tau_f$ value varied from –45 to –76,

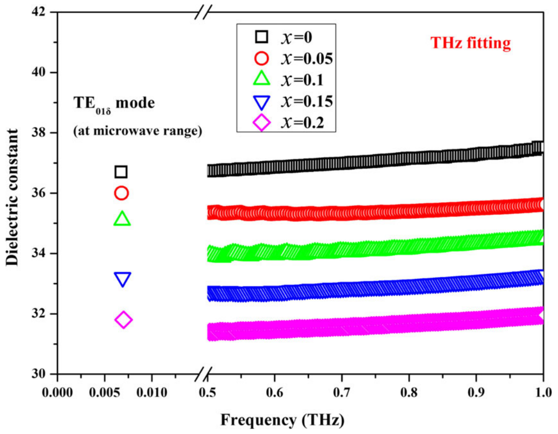

**Fig. 6** Absorption coefficients of $ZnTi_{1-x}(Al_{0.5}Nb_{0.5})_xNb_2O_8$ ceramics at 0.6 and 0.9 THz. Reproduced with permission from Ref. [135], © The American Ceramic Society 2019.

and the $Q\times f$ value was over 95,000 GHz of $MgNb_2O_6$ [140,141]. The investigations about property optimization and preparation methods were concentrated on $MgNb_2O_6$, $ZnNb_2O_6$, and $ZnTa_2O_6$ due to their potential of application. For sintering behavior, the sintering temperature can be reduced to 1150 ℃ of $MgNb_2O_6$ by sol–gel method [142]. Doped ceramics of $(Zn_{1-x}Ni_x)Ta_2O_6$ [143], $Zn(Ta_{1-x}Nb_x)_2O_6$ [144], $Zn(Ta_{1-x}Sb_x)_2O_6$ [145], and composite ceramics composed of $ZnO$–$Nb_2O_5$–$1.75TiO_2$–5 mol% $MgO$, $(1-x)ZnTa_2O_6$–$x MgNb_2O_6$, $(1-x)ZrTi_2O_6$–$x ZnNb_2O_6$, and $(1-x)ZnTa_2O_6$–$x NiNb_2O_6$ were designed successfully to reach near-zero $\tau_f$ value [146–149]. Liu and Deng [150] proposed that the grain size of $ZnNb_2O_6$–$(Zn_{0.7}Mg_{0.3})TiO_3$ ceramics became smaller with the $ZnNb_2O_6$ content increasing. The secondary $ZnV_2O_6$ was observed with higher than 1 wt% $V_2O_5$ into $ZnNb_2O_6$ [151]. The property comparison of $MgTa_2O_6$ was obtained by sol–gel procession and solid reaction sintering by Wu *et al.* [152]. Liu *et al.* [153] verified that the unpaired d-electrons contribution to the room temperature loss should be taken into consideration of $ZrTiO_4$–$ZnNb_2O_6$. It was interesting that the structure transformation was identified as tri-α-$PbO_2$, α-$PbO_2$, trirutile, and rutile in $(1-x)ZnTa_2O_6$–$x TiO_2$ along with the increase of $x$ [154]. $ZnNb_2O_6$ ceramics prepared by microwave sintering exhibited relative density of 94.3%, and the quality factor was dominated by the distribution of grain size [155]. Recently, the intrinsic dielectric properties were investigated using chemical bond theory and lattice vibrational spectra, which indicated that $B_{1u}$ mode at 168.87 $cm^{-1}$ was highly related to the dielectric properties [156], and the fitted results of infrared-related spectrum are presented in Fig. 7.

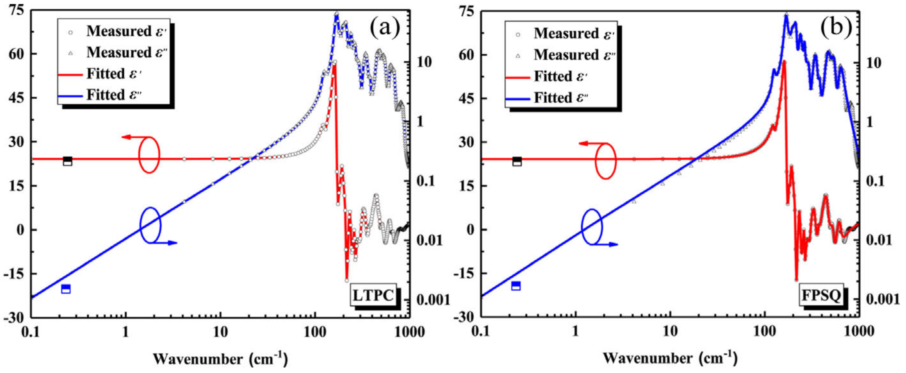

**Fig. 7** Real $\varepsilon'(\omega)$ and imaginary $\varepsilon''(\omega)$ of relative permittivity after (a) LTPC and (b) FPSQ mode fit. LTPC, Lorentz three parameter semiquantum model; FPSQ, four parameter semiquantum model. Reproduced with permission from Ref. [156], © The American Ceramic Society 2019.

### 2.2.2 $ReTiCO_6$ ceramics

The crystal structure of double tantalates of rare-earth elements with titanium tantalite compounds based on $ReTiTaO_6$ is sorted into two parts: orthorhombic aeschynite symmetry with rare earth atomic number in the range of 55–66 and orthorhombic euxenite symmetry with that of 67–71 [157,158]. Generally, high $\varepsilon_r$ and positive $\tau_f$ were obtained for the former, while relatively low $\varepsilon_r$ and negative $\tau_f$ were observed for the latter. The effect of microstructure on properties of $RETiNbO_6$ (RE = La, Sm, and Y) ceramics was presented by Lei *et al.* [159]. The dielectric constant of $RETiNbO_6$ system (RE = Ce, Pr, Nd, Sm, Eu, Gd, Tb, Dy, Y, and Yb) and $RETiTaO_6$ (RE = La, Ce, Pr, Nd, Sm, Eu, Gd, Tb, Dy, Ho, Y, Er, Yb, Al, and In) increases with the RE ionic radius [157,158]. It was reported that $LaTiNbO_6$ usually stabilized as a monoclinic structure, and Zhang and Zuo [160] proposed that ceramics with coexistence of O and M phases could be achieved after prolonging the annealing time. And then, they [161–164] conducted out the substitutions for La and Nb sites, in which the structure evolution, octahedral distortion, and vibrational spectrum were elaborated in detail. More recently, dielectric and optical properties of $Ln_{0.8}Lu_{0.2}TiNbO_6$ (Ln = Ce, Pr, Nd, and Sm) were presented by John and Solomon [165], where the optimal microwave dielectric properties were shown for $Sm_{0.8}Lu_{0.2}TiNbO_6$: $\varepsilon_r \approx 35$, $Q\times f$ value ≈ 37,390 GHz, and $\tau_f \approx 15$ ppm/℃.

### 2.2.3 $ReCO_4/Mg_4C_2O_9/Zn_3C_2O_8$ ceramics

The $ABO_4$ composition material system of $RENbO_4$ (RE = lanthanoid atoms, being La to Lu as well as Y) was firstly investigated in light of their luminescence, damping, and phase transformation characteristics, and their microwave dielectric properties were firstly proposed in 2006 [166]. The satisfied properties of $LaNbO_4$, $NdNbO_4$, and $SmNbO_4$ attracted much attention recently. For $NdNbO_4$ ceramics, substitution for Nd site by single cations such as Sr, Ca, Mn, Co, Mg, Zn, Y, Al, Bi, Sm, La [167–175], and Nb site by Ta, Sb [176–179] were completed to adjust the microwave dielectric properties. In our recent reports, the groups of different isovalent cations of $(A_xB_{1-x})^{5+}$ (A = Mg, Al, Si, Zr; B = W, Mo) have been listed as valid substitution for Nb site to reduce dielectric loss [180–183]. The analysis of combination of P–V–L theory and vibration spectrum suggested that doping into Nb site was beneficial to improving quality factor. Meanwhile, $NdNbO_4$ prepared in sol–gel method or composite ceramics composed of $NdNbO_4$–$CaTiO_3$ [184], $NdNbO_4$–$CaF_2$ [185], and $NdNbO_4$–MgO [186] have also been reported to perfect the properties. Similarly, intrinsic dielectric properties of $EuNbO_4$ were studied by Liu *et al.* [187]. In the full range of $La_2O_3$–$Nb_2O_5$–$V_2O_5$ system, four typical phase regions were verified, including monoclinic fergusonite, tetragonal sheelite, B-site ordered sheelite, and composite of monoclinic $LaVO_4$ and tetragonal sheelite phases [188]. Likewise, MgO was designed as an addition for $LaNbO_4$ ceramics and the excellent properties were listed as $\varepsilon_r \approx 19.8$, $Q\times f$ value ≈ 94,440 GHz, and $\tau_f \approx$ 6.1 ppm/℃ [189]. More recently, structure–property relationship of another $A^{3+}B^{5+}O_4$ binary oxide, zircon-type $AVO_4$ (A = Eu, Y) ceramics, was discussed by packing fraction and bond valence [190]. Ferroelastic phase transition from monoclinic fergusonite to tetragonal scheelite was observed by *in situ* Raman spectroscopy and X-ray diffraction of $La(Nb_{0.9}V_{0.1})O_4$, and the schematic of $\varepsilon_r$ typical-ceramics versus temperature was shown by Zhou *et al.* [191]. NiO/CoO added into $LaNbO_4$ would distinctly optimize the quality factor since the larger and uniform grain was obtained [192]. Although the thermal properties [193–196] and the first-principles calculation of electronic structure and optic properties of $RETaO_4$ (RE = Y, La, Sm, Eu, Dy, Er) [197] have been investigated, the intrinsic dielectric loss has not been summarized in this system. Microwave dielectric properties of $ErNbO_4$ prepared by sol–gel method were reported by Devesa *et al.* [198], and the grain size varied from 31.27 to 86.65 μm and 40.96 to 78.23 μm by Rietveld refinement and Sherrer's formula, respectively. $ZrTiO_4$ followed the general formula of $ABO_4$, and the intrinsic dielectric loss of $Zr_{0.8}Sn_{0.2}TiO_4$ was investigated by THz time domain spectroscopy [199].

The structure of corundum-like phase of $Mg_4Nb_2O_9$ was verified by Kumada *et al.* [200], where the cations were ordered by the stack of two layers of a mixture of Mg and Nb and one layer of Mg along the *c*-axis. $Mg_4(Nb_{2-x}Ta_x)O_9$ solid solution was synthesized in the sintering temperature range of 1350–1400 ℃ [201], which possesses a comparable quality factor ($Q\times f$ value ≈ 350,000 GHz for $x = 2$) to that of $Al_2O_3$. To deal with the limitation of high sintering temperature, both $Mg_4Nb_2O_9$ and $Mg_4Ta_2O_9$ were generated by sol–gel method and the variation of property with sintering temperature was analyzed [202–204]. An accompanying minor phase of $Mg_5Nb_4O_{15}$ gradually disappeared as the calcined temperature increased to 850 ℃. High frequency dielectric properties of $A_5B_4O_{15}$ microwave dielectric were evaluated by Kamba *et al.* [205] using far-infrared reflection, transmission spectroscopy, and time-resolved THz transmission spectroscopy. Considering the negative influence of second phase on properties and sintering behavior of $Mg_4Nb_2O_9$, excess MgO and $Mg(OH)_2$ were used to adjust the composition of $Mg_4Nb_2O_9$ [206,207], which presented that the appearance of $Mg_4Nb_2O_9$ pure phase was more easily with $Mg(OH)_2$ as raw materials. A dramatically improvement of quality factor was achieved by Ni and Ta co-doped into this system, and $(Mg_{0.95}Ni_{0.05})_4(Nb_{1-x}Ta_x)_2O_9$ shows satisfied properties of $\varepsilon_r \approx 12.76$, $Q\times f$ value ≈ 442,000 GHz, and $\tau_f \approx -54$ ppm/℃, when $x = 1$ and sintered at 1375 ℃ [208]. $(B_xW_{1-x})^{5+}$ substitution at $Nb^{5+}$ site (B = Li, Mg, Al, Ti) in $Mg_4Nb_2O_9$-based ceramics revealed that the $\tau_f$ depended on the distortion of the oxygen octahedra, while $(Ti_{1/2}W_{1/2})^{5+}$ substitution had the highest quality factor of 233,000 GHz [209]. The investigation of $y(Mg_{0.95}Co_{0.05})_4Ta_2O_9$–$(1-y)CaTiO_3$ ceramics provided a promising dielectric material for application with temperature stability, and the properties were shown as $\varepsilon_r \approx 25.78$, $Q\times f$ value ≈ 200,000 GHz, and $\tau_f \approx -4.69$ ppm/℃ [210].

$Zn_3Nb_2O_8$ was another promising binary niobite compound, which could be successfully produced with 98% theoretical density sintered at 1100 ℃ [211]. A two-stage sintering method was proposed to optimize the microstructure of $Zn_3Nb_2O_8$ [212], where the sintering temperatures were 1150 and 1200 ℃ for the first time and the second sintering temperatures were 1050 and 1100 ℃, respectively. Sintered based on this approach, ceramics presented denser grain packing and less abnormal grain growth. Adding secondary phase into ceramics to compensate for $\tau_f$ value would introduce a large amount of second phase, which were ascribed to the large dielectric loss. Aiming to reduce the defects stemmed from the secondary phase, layer-cofired ceramic architectures were designed such as $Zn_{1.01}Nb_2O_6/TiO_2/Zn_{1.01}Nb_2O_6$ [3], $MgTiO_3/TiO_2/MgTiO_3$ [4], and $Zn_3Nb_2O_8/TiO_2/Zn_3Nb_2O_8$ [5]. High-$Q$ value was remained and temperature-stable MWDCs were obtained for all the reported tri-layer co-fired ceramics.

## 2. 3 Rock-salt structure

Closely followed by the ever-growing explosion of global data volume and the rapid boost of millimeter-wave technology, the requirement of materials with low permittivity ($\varepsilon_r \leqslant 25$) and high $Q\times f$ value is increasingly urgent. In the exploration of new composition ceramics, many rock-salt Li-containing compounds emerge as focal points. The general formula of rock-salt ceramics is $A_aB_bO_{a+b}$ ($A^+$ = Li, Na; $B^{4+}$ = Ti, Sn, Zr; $B^{5+}$ = Nb and Ta). $Li_2TiO_3$ underwent an order–disorder phase transition at 1213 ℃, in which the structure consisted of ordered (Li,Ti) layer, with the property of $\varepsilon_r \approx 12.76$, $Q\times f$ value ≈ 44,200 GHz, and $\tau_f \approx -54$ ppm/℃ [213]. The sintering behavior of excess Li for non-stoichiometry $Li_{2+x}TiO_3$ ceramics was investigated by Bian and Dong [214] and Hao *et al.* [215] after the determination of pseudo-binary of $Li_2O$–$TiO_2$ [216,217]. For co-doped substitution, $Zn_{1/3}Nb_{2/3}$, $Mg_{1/3}Nb_{2/3}$, and $Co_{1/3}Nb_{2/3}$ addition into $Li_2TiO_3$ could adjust the $\tau_f$ from positive to negative [218–220]. $Cu_{1/3}Nb_{2/3}$ doped ceramics with 3 wt% $H_3BO_3$ were designed as a patch antenna and a dielectric resonator antenna [221]. The solid solution of $Li_2TiO_3$–MgO [222], $Li_2TiO_3$–ZnO [223,224], and $Li_2TiO_3$–$Li_3NbO_4$ [225] attracted much interest of researcher owing to their high quality factor. The primarily reported ceramics of $Li_2O$–MgO/ZnO/CoO–Ti/Sn/$ZrO_2$ ternary system contain $Li_2Mg/NiTi/ZrO_4$, $Li_2Zn/Mg/CoTi_3O_8$, $Li_2Zn/Co/Mg_3Ti_4O_{12}$, $Li_2Mg/Ni_3Ti/SnO_6$, $Li_2ZnTi_5O_{12}$, $Li_2Mg_4TiO_7$, $Li_6Mg_7Ti_3O_{16}$, $Li_4MgSn_2O_7$, and $Li_2NiZrO_4$; while $LiZnNbO_4$, $Li_3Mg_2NbO_6$, and $Li_2Mg_3NbO_6$ occupied the dominated composition of $Li_2O$–MgO/ZnO/CoO–Nb/Ta/$Sb_2O_5$. The microwave dielectric properties of the mentioned pure phase ceramics are listed in Table 1 [224,226–246], and the phase diagram of rock-salt structure is plotted in Fig. 8, where the ordered–disordered range was summarized from Zhang *et al.* [246,247]. Simultaneously, Gu *et al.* [248] stated the two-phase and thermally stable ceramics of $0.8Li_3NbO_4$–$0.2Ca_{0.8}Sr_{0.2}TiO_3$, where the $\tau_f$ value was 5.2 ppm/℃.

### 2.3.1 *$Li_2O$–MgO/ZnO/CoO–Ti/Sn/$ZrO_2$ ternary system*

Secondary phases of $Mg_2TiO_4$ and $Li_2Mg_3Ti_4O_{12}$ were highly related to the properties when Yao *et al.* [249] prepared the $Li_2MgTiO_4$ after sintered higher than 1250 ℃. The variation of dielectric properties of $Li_2Mg_{0.95}A_{0.05}TiO_4$ (A = Ni, Co, Mn, Zn) indicated that the dielectric polarizability dominated the dielectric constant [250], and a near zero $\tau_f$ (–4.03 ppm/℃) was obtained for 0.1 mol Zn substitution for Mg [251].

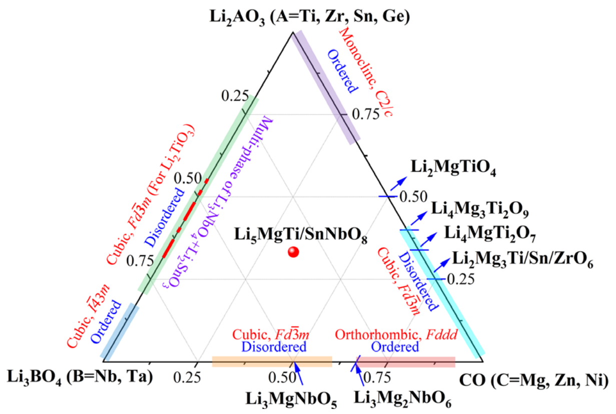

**Fig. 8** Pseudo phase diagram of rock-salt structure.

**Table 1 Microwave dielectric properties of pure phase with rock-salt structure**

| Formula | $\varepsilon_r$ | $Q\times f$ (GHz) | $\tau_f$ (ppm/℃) | ST (℃) | Ref. |
|---|---|---|---|---|---|
| $Li_2MgTiO_4$ | 17.25 | 97,300 | –27.2 | 1360 | [226] |
| $Li_2NiTiO_4$ | 19.25 | 51,290 | –20.1 | 1275 | [230] |
| $Li_2MgZrO_4$ | 12.30 | 40,900 | –12.31 | 1175 | [234] |
| $Li_2MgTi_3O_8$ | 27.2 | 40,000 | 2.6 | 1000 | [227] |
| $Li_2CoTi_3O_8$ | 28.9 | 52,600 | 7.4 | 1025 | [228] |
| $Li_2ZnTi_3O_8$ | 25.6 | 90,000 | –10.8 | 1000 | [229] |
| $Li_2Co_3Ti_4O_{12}$ | 21.4 | 35,000 | –22 | 1050 | [233] |
| $Li_2Mg_3Ti_4O_{12}$ | 20.2 | 62,300 | –27.1 | 1125 | [232] |
| $Li_2Zn_3Ti_4O_{12}$ | 20.6 | 106,700 | –48 | 1075 | [231] |
| $Li_2Mg_3TiO_6$ | 15.2 | 152,000 | –39 | 1280 | [235] |
| $Li_2Ni_3TiO_6$ | 13.18 | 9800 | –7.3 | 1275 | [238] |
| $Li_2Mg_4TiO_7$ | 13.43 | 233,600 | –7.24 | 1600 | [239] |
| $Li_6Mg_7Ti_3O_{16}$ | 15.27 | 209,400 | –11.31 | 1550 | [240] |
| $Li_2ZnTi_5O_{12}$ | 38.4 | 54,300 | 82.9 | 1260 | [237] |
| $Li_4MgSn_2O_7$ | 12.4 | 58,754 | 12.1 | 1180 | [241] |
| $Li_2NiZrO_4$ | 12.3 | 20,000 | –23.4 | 1300 | [242] |
| $LiZnNbO_4$ | 15.6 | 85,310 | –63.7 | 1070 | [236] |
| $Li_3Mg_2NbO_6$ | 14.94 | 100,965 | –21.96 | 1225 | [243] |
| $Li_3Mg_2SbO_6$ | 10.5 | 84,600 | –9.0 | 1300 | [244] |
| $Li_2Mg_3NbO_6$ | 16.8 | 79,642 | –22 | 1300 | [224] |
| $Li_3MgNbO_5$ | 16.2 | 96,796 | –24.8 | 1260 | [245] |

ST: sintering temperature (℃).

Both $Li_2Mg_4TiO_7$ and $Li_4Mg_3Ti_2O_9$ exhibited $LiFeO_2$-like cubic phase with space group $Fm\bar{3}m$. The optimal combination of microwave dielectric properties of $Li_2(Mg_{0.9}A_{0.1})_4TiO_7$ (A = Co, Ni, Mg, Zn, Ca) was observed for Zn doped ceramics ($\varepsilon_r \approx 14.77$, $Q\times f$ value ≈ 162,200 GHz, and $\tau_f \approx -4.30$ ppm/℃) and Ca ($\varepsilon_r \approx 15.79$, $Q\times f$ value ≈ 100,300 GHz, and $\tau_f \approx -1.43$ ppm/℃) [252]. Pure cubic $Li_4Mg_3Ti_2O_9$ phase was formed in the whole range of $0 \leqslant x \leqslant 0.4$ with $Mg_{1/3}Ta_{2/3}$ occupying Ti site [253].

Except for the sintering temperature, the heating rates and substation will directly influence the grain size, densification, and properties. Lu *et al.* [254] pointed out that the sintering rate increasing from 3 to 7 ℃/min would deteriorate the quality factor of $Li_2ZnTi_3O_8$ ceramics. If ball milling is applied for the raw materials at first for 4 h, then the sintering temperature of $Li_2ZnTi_3O_8$ ceramics could reduce from 1075 to 925 ℃, and those ceramics were chemically compatible with Ag [255]. Sintering the ceramics in a box type electric furnace and in a microwave furnace would obtain $Li_2ZnTi_3O_8$ ceramics with the grain size of 38 and 7 μm, respectively [256]. Mg, Co, and Zn substitution for Zn in $Li_2ZnTi_3O_8$ increased the quality factor because of the more compact microstructure [257–259]. Whereas, the secondary phases were recorded after the introduction of $Sr^{2+}$ or $(Sr_xCa_{1-x})$ into $Li_2ZnTi_3O_8$ [260–262]. Phase evolution of $(1-x)Li_2ZnTi_3O_8-xTiO_2$ system indicated that pure $Li_2ZnTi_3O_8$ with cubic structure was observed when $x \leqslant 0.2$ (the lattice parameter is similar to $MgFe_2O_4$ with space group of $Fm\bar{3}m$ (227)), solid solution was exited in the range of $0.2 \leqslant x \leqslant 0.4$ with cubic structure (the lattice parameters is similar to $Zn_2Ti_3O_8$ with space group of $P4332$ (212)), and rutile $TiO_2$ phase appeared when $x \geqslant 0.6$ [263]. The $\tau_f$ value moves from –15 to 102.4 in $(1-x)Li_2ZnTi_3O_8-xTiO_2$ ($0 \leqslant x \leqslant 0.4$) [264]; meanwhile, near zero $\tau_f$ value was also achieved by Bari *et al.* [265] in this system. 4 wt% $TiO_2$ was added into $Li_2ZnTi_3O_8$ with different particle sizes, where the nanoparticles and micron particles all generated a more uniform microstructure and relative density reached to 98.5% [266]. Similar to $TiO_2$-doped $Li_2ZnTi_3O_8$ ceramics, phase composition and properties of $Li_2Mg(Ti_{1-x}Sn_x)_3O_8$ ($x$ = 0.1–0.25) were concluded as with $0.10 \leqslant x \leqslant 0.15$, the spinel and rutile were co-exited; with $0.20 \leqslant x \leqslant 0.25$, the spinel, rutile, and ilmenite were obtained [267], and the optimal properties of $Li_2ZnTi_3O_8$–$0.2SnO_2$ composite ceramics exhibited: $\varepsilon_r \approx 20.9$, $Q\times f$ value ≈ 89,500 GHz, and $\tau_f \approx -24$ ppm/℃ [268]. The variation of dielectric properties with density of $(1-x)Li_2(Mg_{0.95}Zn_{0.05})_3Ti_3O_8-xLi_2TiO_3$ ($x$ = 0.727, 0.778, 0.821, and 0.889) was discussed systematically by Zhang *et al.* [269]. The concentration of oxygen vacancy, relative density, and decrease in damping behavior would influence the $Q\times f$ value of $Li_2ZnTi_3O_8$–$x$ wt% $Nb_2O_5$ [270]. To trace the dielectric response of lattice vibration, the response process of dielectric loss in $Li_2ZnTi_{3-x}M_xO_8$ (M = $Al^{3+}$, $Nb^{5+}$, $(Al_{0.5}Nb_{0.5})^{4+}$,

$(Zn_{1/3}Nb_{2/3})^{4+}$, and $(Li_{1/4}Nb_{3/4})^{4+}$) was discussed systematically containing the conduction loss and lattice vibration loss [271]. The conduction loss which acts at frequency lower than terahertz is neglectful by researchers concentrating on MWDCs, while AC impedance analysis was used to identify the effect of dopants and the mechanism of conduction loss in this system. Combining the fitting THz time domain spectrum and far infrared reflectivity spectrum, the dielectric response was illustrated in depth based on lattice loss and conduction loss.

Ultra-low loss microwave dielectric materials of $Li_2Mg_3TiO_6$-based ceramics are extensively studied via doping cations into Mg and Ti site. Bivalent cations [272] such as $Ca^{2+}$, $Ni^{2+}$, $Zn^{2+}$, and $Mn^{2+}$ were verified effectively to adjust the microwave dielectric properties for Mg site, and co-doped of $Al_{1/2}Nb_{1/2}$ and $Zn_{1/3}Nb_{2/3}$ for Ti-site enhanced the $Q\times f$ values to 174,300 GHz [273] and 168,911 GHz [274], respectively. For MWDCs, compactness microstructure guarantees the satisfied microwave dielectric properties. However, porous microstructure caused by the loss of Li element under high temperature is a problem for all compounds containing Li. To cure the volatilization of lithium, Fang *et al*. [275–277] proposed a reliable method which provided the Li-rich sintering atmosphere, and they obtained serial MWDCs based on Li–Mg–Sn/Ti oxides with excellent properties. The schematic representation of the devices provided with the Li-rich atmosphere is shown in Fig. 9, and this similar method was gradually popularized to other systems with volatilization element to obtain the ceramics with dense microstructure. The negative $\tau_f$ values can be compensated by $Ca_{0.8}Sr_{0.2}TiO_3$, and the sample with $0.91Li_2Mg_3TiO_6–0.09Ca_{0.8}Sr_{0.2}TiO_3$ showed a $\tau_f$ value of –3.65 ppm/℃ [278].

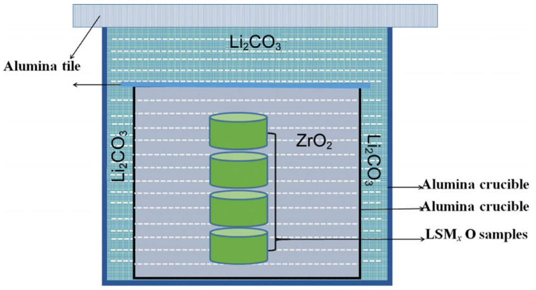

**Fig. 9** Schematic representation of the $Li_{2/3(1-x)}Sn_{1/3(1-x)}Mg_xO$ ($x$ = 0–4/7) placement for providing $ZrO_2$-burying protective atmosphere and Li-rich sintering atmosphere. Reproduced with permission from Ref. [277], © The American Ceramic Society 2017.

The phase evolution of $Li_2O–3MgO–mTiO_2$ ($1 \leqslant m \leqslant 6$) was summarized as the phase diagram shown in Fig. 10 [279], where the phase structures changed as ($Li_2Mg_3TiO_6$, $m$ = 1)→($Li_2Mg_3Ti_4O_{12}$ and $Mg_2TiO_4$, $m$ = 2, 3)→($Li_2Mg_3Ti_4O_{12}$, $m$ = 4)→($Li_2Mg_3Ti_4O_{12}$, $MgTiO_3$, and $Li_2MgTi_3O_8$, $m$ = 5)→($Li_2Mg_3Ti_4O_{12}$, $MgTiO_3$, $Li_2MgTi_3O_8$, and $MgTi_2O_5$, $m$ = 6). The application of P–V–L theory to $Li_2MgTiO_4$ [280], $Li_4Mg_3Ti_2O_9$ [281], and $Li_2Mg_3TiO_6$ [282] revealed that the bond ionicity ($f_i$) descended as $f_i$(Ti–O) > $f_i$(Mg–O) > $f_i$(Li–O). As analogy with $Li_2O–3MgO–mTiO_2$ ceramics, $Li_2ZrO_3–MgO$ ceramics were explored as well [234,283,284]. High quality factor could be obtained in $(Mg_{1/3}Sb_{2/3})^{4+}$ substitutions for $Li_2Mg_4ZrO_7$ ceramics, which reached 153,140 GHz [285]. Zirconium deficiency of $Li_2Mg_3Zr_{1-x}O_6$ ceramics was designed and remarkable dielectric properties were presented: $\varepsilon_r \approx 13.13$, $Q\times f$ value ≈ 116,400 GHz, and $\tau_f \approx -26.30$ ppm/℃ [286].

### 2.3.2 *$Li_2O–MgO/ZnO/CoO–Nb/Ta_2O_5$ ternary system*

An intermediate compound of $Li_3Mg_2NbO_6$ at $x$ = 1/3 appeared in the investigation of structure evolution of $Li_{(3-3x)}M_{4x}Nb_{(1-x)}O_4$ (M = Mg, Zn), and the results indicated that solid solution could be formed in a wide range between $Li_3NbO_4$ and MgO [224]. Considering the existed compounds of $Li_2TiO_3–MgO$ and $Li_3NbO_4–MgO$, Zhang *et al*. [246] supposed that a three-component solid solution would be formed in $Li_2TiO_3–Li_3NbO_4–MgO$, and the pseudo phase diagrams of those component were presented in Fig. 11. The most extensively studied ceramics in this system are $Li_2Mg_3Nb/TaO_6$ and $Li_3Mg_2NbO_6$. For instance, a large grain size (130 μm) was recorded after using reaction sintering process to generate $Li_3Mg_2NbO_6$ [287]. Single cation doped solid solution of $Li_3(Mg_{1-x}Co_x)_2NbO_6$ [288], $Li_3(Mg_{1-x}Mn_x)_2NbO_6$ [289], $Li_3Mg_2Nb_{1-x}Mo_xO_{6+x/2}$ [290],

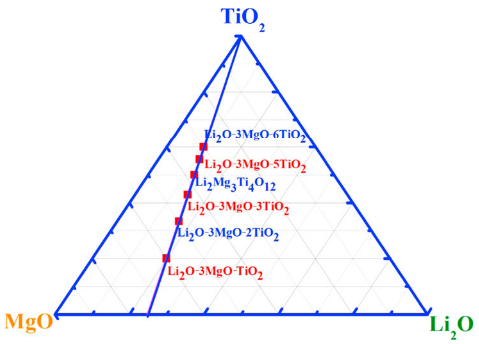

**Fig. 10** Ternary phase diagram of $Li_2O–3MgO–mTiO_2$ systems. Reproduced with permission from Ref. [279], © Elsevier Ltd and Techna Group S.r.l. 2016.

$Li_3Mg_2Nb_{1-x}Ta_xO_6$ [291], $Li_3Mg_2Nb_{1-x}V_xO_6$ [292], $Li_3Mg_2Sb_{1-x}O_6$ [293], $Li_3Mg_{2-x}Zn_xSbO_6$ [294], $Li_3Mg_2(Nb_{1-x}W_x)O_{6+x/2}$ [295], $Li_3Mg_2Nb_{1-x}Ti_xO_{6-x/2}$ [296], $Li_{3+x}Mg_2Nb_{1-x}Ti_xO_6$ [297], and $Li_3Mg_2Nb_{0.96}(M_xW_{1-x})_{0.04}O_6$ (M = $Li^+$, $Mg^{2+}$, $Al^{3+}$, $Ti^{4+}$) [298] or non-stoichiometric $Li_3Mg_{2+x}SbO_6$ [299] have been probed and analyzed to explain the variation of dielectric properties through current theory including P–V–L theory, packing fraction, and C–M equations. It was interesting that the "dark hole" phenomenon of $Li_2TiO_3$ was cured by adding $Li_3Mg_2NbO_6$ and the $\tau_f$ value of $0.96Li_2TiO_3$–$0.04\ Li_3Mg_2NbO_6$ was 2.6 ppm/℃ [300]. Since yet there was no literature about the structure transformation of $Li_2TiO_3$–$Li_3NbO_4$–MgO to renew the understanding of rock-salt ceramics, Zhang *et al*. [247,301,302] gradually updated the reports about $Li_3Mg_2NbO_6$-based ceramics. The phase transitions among the orthorhombic, cubic, and monoclinic were verified by XRD (Fig. 12) and TEM analysis (Fig. 13). The systematical analysis of lattice evolution and ordering transformation indicated that the low dielectric loss of this system was mainly ascribed to the superlattice. The THz time-domain spectroscopy was firstly used in this system to evaluate the intrinsic dielectric loss associated with phonon oscillation. Meanwhile, the configurational entropy was calculated to explain the change of disordered and ordered crystal structures, where the disordering cubic phase generated much larger configurational entropy than the ordered orthorhombic and monoclinic phase (Fig. 14).

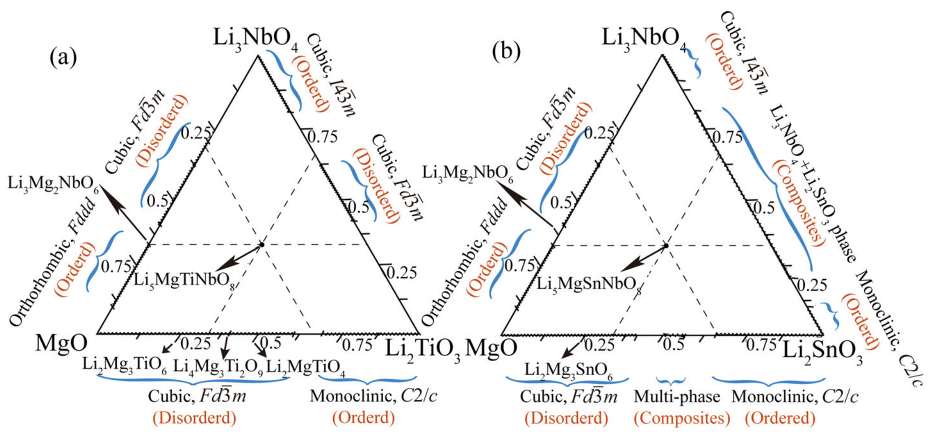

**Fig. 11** Pseudo phase diagrams of (a) $Li_2TiO_3$–$Li_3NbO_4$–MgO and (b) $Li_2SnO_3$–$Li_3NbO_4$–MgO ternary systems. Reproduced with permission from Ref. [246], © Elsevier Ltd and Techna Group S.r.l. 2020.

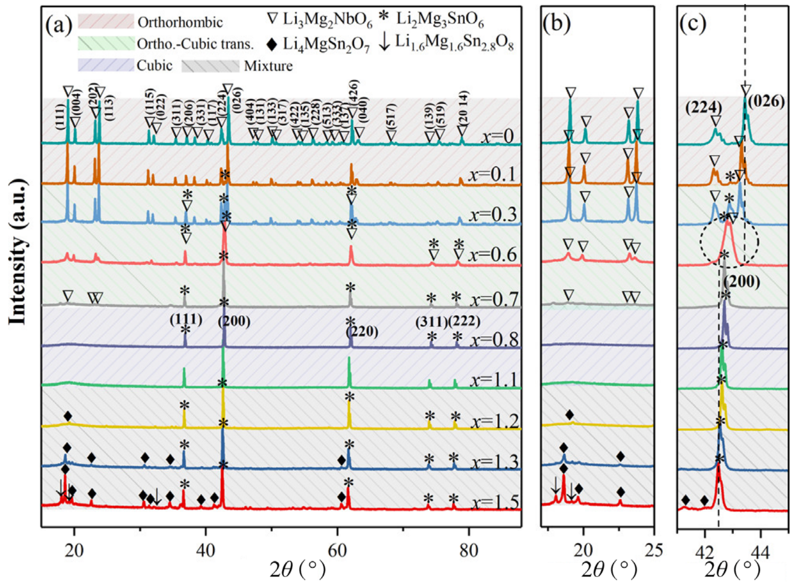

**Fig. 12** (a) XRD patterns of $Li_3Mg_{2-x/3}Sn_xNb_{1-2x/3}O_6$ ($x$ = 0–1.5) MWDCs sintered at 1290 ℃ for 4 h. (b) Amplified spectra of the XRD patterns from 17° to 25°. (c) Amplified spectra of the XRD patterns from 41° to 45°. Reproduced with permission from Ref. [302], © American Chemical Society 2020.

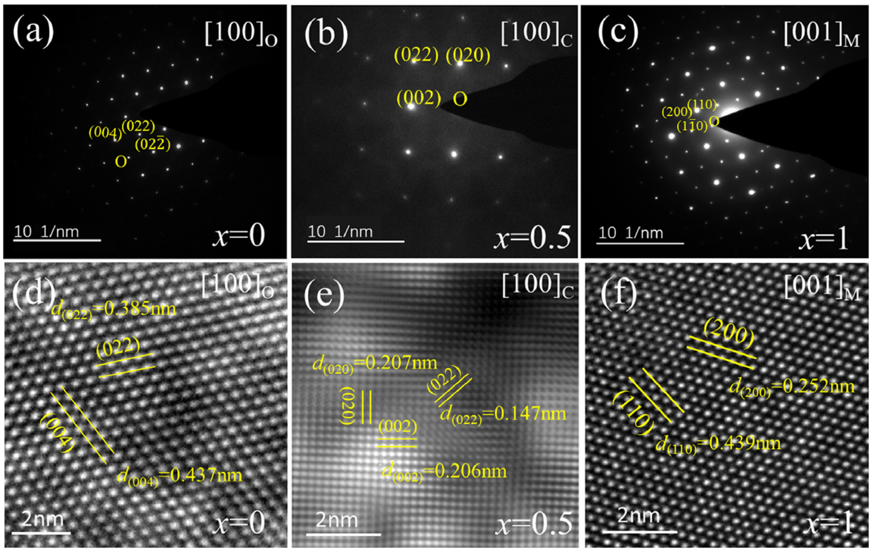

**Fig. 13** Selected area electron diffraction (SAED) patterns of $Li_{3+x}Mg_{2-2x}Nb_{1-x}Ti_{2x}O_6$ ($0 \leqslant x \leqslant 1$) ceramics for (a) $x = 0$ sample taken along $[100]_O$ zone axis, (b) $x = 0.5$ sample along $[100]_C$ zone axis, and (c) $x = 1$ sample along $[001]_M$ zone axis. (d–f) Corresponding high resolution transmission electron microscopy (HRTEM) images of the selected areas of the above samples. Reproduced with permission from Ref. [247], © Elsevier Ltd and Techna Group S.r.l. 2020.

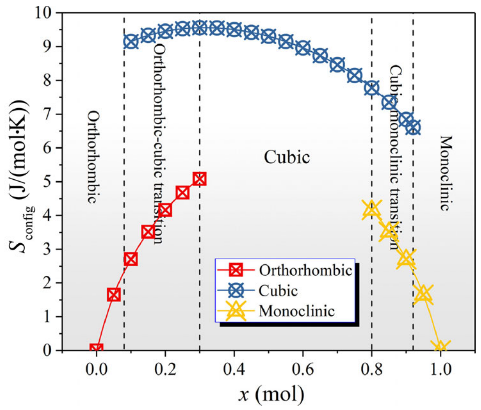

**Fig. 14** Configurational entropy ($S_{config}$) of the cation substitutions for the three different types of phases in the $Li_{3+x}Mg_{2-2x}Nb_{1-x}Ti_{2x}O_6$ ($0 \leqslant x \leqslant 1$) system as a function of the substitution amount ($x$). Reproduced with permission from Ref. [247], © Acta Materialia Inc. 2021.

In contrast to the large scale studies of $Li_3Mg_2NbO_6$-based ceramics, only $Li_4MgSn_{(2-1.25x)}Nb_xO_7$ ($0 \leqslant x \leqslant 0.15$) was reported to evaluate the change of microstructure in $Li_4MgSn_2O_7$. The mean grain size of the doped ceramics ranged from 1.35 to 4.01 μm and the cracks appeared along with the occurrence of the secondary phase [303].

## 2.4 Tungsten bronze structure and titanate based on BaO–TiO$_2$

Since 1970, the exploration of BaO–TiO$_2$ system has been continuous renewed. Among them, BaO–4TiO$_2$ and 2BaO–9TiO$_2$ are the most extensively investigated ceramics as the representative ceramics with medium dielectric constant. The pseudo phase diagram of tungsten bronze structure and binary system based on BaO–TiO$_2$ system is shown in Fig. 15. In contrast to other sections in this review, the investigations about the compounds within this phase diagrams are relatively less, because the study of ceramics in BaO–$R_2O_3$–TiO$_2$ (R = La–Gd) has been almost accomplished and widely used in the industry.

### 2.4.1 *BaO–TiO$_2$/Nb$_2$O$_5$/Ta$_2$O$_5$ system and Re$_2$TiO$_5$*

The frequency dependence of $Q{\times}f$ value was observed for $Ba_2Ti_9O_{20}$ ceramics, which was ascribed to the extrinsic dielectric loss [304]. After adding $Sm_2O_3$ into $BaTi_4O_9$, precursor of $BaTi_4O_9$ and $BaSm_2Ti_4O_{12}$ was

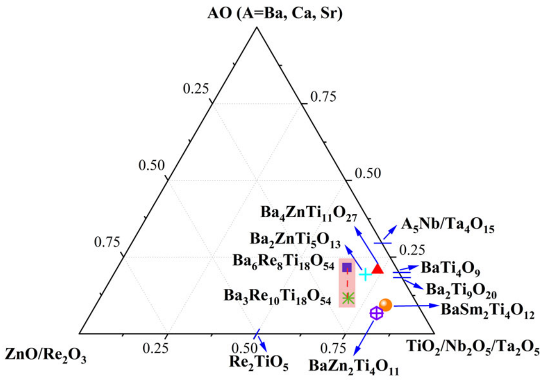

**Fig. 15** Pseudo phase diagram of tungsten bronze structure and binary titanite.

modeled by a cool iso-static press and calcined at 1300 ℃, and a near-zero temperature coefficient of +2.2 ppm/℃ was achieved with 40 mol% $Sm_2O_3$ [305]. $(Zn_{1/3}Nb_{2/3})^{4+}$ substitution for $Ti^{4+}$ in $Ba_2Ti_9O_{20}$ modified the $\tau_f$ value to +7 ppm/℃ [306]. Pseudobrookite-type $A_5B_4O_{15}$ (A = Ba, Sr, Mg, Ca; B = Nb, Ta) was firstly investigated by Jawahar *et al.* [307], which showed $\varepsilon_r \approx$ 11–51, $Q\times f$ value ≈ 2400–88,000 GHz, and $\tau_f \approx$ (–73)–232 ppm/℃. Based on sol–gel method, $Mg_5Nb_4O_{15}$ nano-powders were obtained at 600 ℃, and then the sintering temperature can be reduced to 1300 ℃ [204]. On the basis of P–V–L chemical bond theory, the relationship of chemical bond characteristic and microwave dielectric properties of $Eu_2TiO_5$ was discussed deeply [308]. Meanwhile, the electron localization function (ELF) based on the first-principles calculation was evaluated to provide the information of bond covalency [309], which provided a strategy to estimate the chemical bond characterization.

### 2.4.2 *Tungsten bronze structure*

The different compositions of tungstenbronze-type with $Ba_{6-3x}R_{8+2x}Ti_{18}O_{54}$ solid solution reported by Ohsato [310] in 2001, and the compounds were presented in Fig. 16. The relative permittivity of $BaO–R_2O_3–TiO_2$ (R = La–Gd) was higher than 80, and the solid solubility region became narrower as the ionic radius of rare earth increasing [311]. The doping effect and the determination of crystal structure of $Ba_{6-3x}R_{8+2x}Ti_{18}O_{54}$ were summarized in the review of dielectric materials for wireless communication [1]. After 2010, there are only a few studies focused on this system. Three distinct phases were formed using variable size $TiO_2$ reagents into $BaO–Nd_2O_3–TiO_2$ [312]. $Ba_{6-3x}R_{8+2x}Ti_{18}O_{54}$ (BRT, R = La, Pr, Nd, Sm)

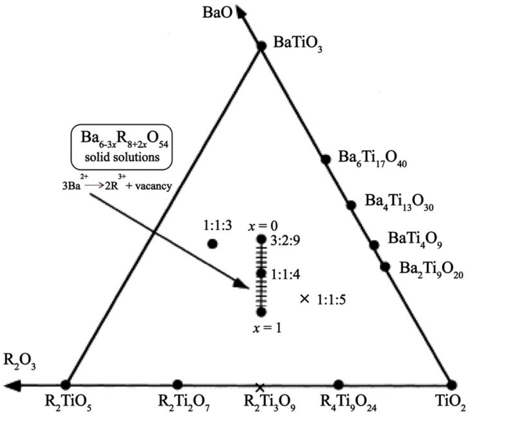

**Fig. 16** $BaO–R_2O_3–TiO_2$ (R = rare earth) ternary system. Reproduced with permission from Ref. [310], © Elsevier Science Ltd. 2001.

solid solution family was reported with high permittivity. When $x$ = 2/3, $Ba_4Nd_{9.33}Ti_{18}O_{54}$ was regarded as the most investigated ceramics to lower its $\tau_f$ value and sintering temperature or improve its $Q\times f$ value. Yao *et al.* [313] and Chen *et al.* [314] proposed that with $Al_2O_3$ added $BaO–Nd_2O_3–TiO_3$ ceramics, the $Q\times f$ value would increase obviously. The temperature-stable ceramics could be obtained by Pb and Sr substitution for $Ba_{3.75}Nd_{9.5}Ti_{18}O_{54}$ [315]; $Ba_{4.5}Re_9Ti_{18}O_{54}$ (Re = La, Nd) [316]; solid solution of $Ba_{4.2}Nd_{9.2}Ti_{18-x}Sn_xO_{54}$ [317], $(Ba_{0.98}Sr_{0.02})_{3.75}Nd_{9.5}Ti_{18x}(Zn_{1/3}Nb_{2/3})_xO_{54}$ [318], $Ba_{6-3x}Nd_{8+2x}Ti_{18-y}(Cr_{1/2}Nb_{1/2})_yO_{54}$ [319], $Ba_4Nd_{9.33}(Al_{0.5}Nb_{0.5})_xTi_{18-x}O_{54}$ [320], $Ba_{3.75}Nd_{9.5}Ti_{18-z}(Al_{1/2}Nb_{1/2})O_{54}$ [321], and $Ba_xSr_{1-x}TiO_3$ [322]; $NdAlO_3$ [323] addition to $Ba_4Sm_{9.33}Ti_{18}O_{54}$; MgO, $Al_2O_3$, and $MnO_2$ substituted for $Ti^{4+}$ [324] in $Ba_{4.2}Sm_{9.2}Ti_{18}O_{54}$, $Ba_4La_{3.73}Sm_{4.66}Bi_{0.93}Ti_{18}O_{54}$, $Ba_4(Pr_{0.4}Sm_{0.6})_{28/3}Ti_{18-y}Ga_{4y/3}O_{54}$ [325], and $Ba_4(Pr_{1-x}Sm_x)_{28/3}Al_{4y/3}O_{54}$ [326]. Among those reports, the analysis of Raman spectrum of $Ba_{3.75}Nd_{9.5}Ti_{18-z}(Al_{1/2}Nb_{1/2})O_{54}$ enriched the theoretical study of tungstenbronze-type.

### 2.4.3 *BaO–ZnO–TiO₂ system*

Ceramics based on the $BaO–ZnO–TiO_2$ system have been concluded as $Ba_4ZnTi_{11}O_{27}$, $BaZn_2Ti_4O_{11}$, $Ba_2ZnTi_5O_{13}$, and $Ba_xZn_xTi_{8-x}O_{16}$–hollandite [327]. The substitution of Cu for Zn dramatically increased the $Q\times f$ value because of the restrain of the formation of $Ti^{3+}$ ions [328]. Considering the opposite $\tau_f$ values of $BaTi_4O_9$ and $BaZn_2Ti_4O_{11}$, the $\tau_f$ values of composite ceramic based on those two phases varied gradually from 12 to –13 ppm/℃ [329]. CuO also worked as flux former to enhance the densification in the $BaTi_4O_9$–$BaZn_2Ti_4O_{11}$ composite ceramics, and the $0.85BaTi_4O_9$–$0.15BaZn_2Ti_4O_{11}$–1 wt% CuO presented the properties as $\varepsilon_r \approx$ 36.4, $Q\times f$ value ≈ 62,600 GHz, and $\tau_f \approx$ +0.2 ppm/℃ [330]. Phase evolution of $BaZn_2Ti_4O_{11}$–$BaNd_2Ti_4O_{12}$ ceramics was determined by Yu *et al.* [331], where $0.8BaZn_2Ti_4O_{11}$–$0.2BaNd_2Ti_4O_{12}$ ceramics possessed properties as $\varepsilon_r \approx 39.1$, $Q\times f$ value ≈ 37,850 GHz, and $\tau_f \approx -9$ ppm/℃.

## 2.5 Perovskite related structure

The ideal perovskite (written as $ABO_3$) is cubically symmetric with a space group of $Pm\overline{3}m$, and the represented material is $SrTiO_3$. Due to the flexibility of $ABO_3$ perovskite, variants of perovskite have been investigated, and the classification of perovskite-related structure with representative structure is summarized in Fig. 17. The perovskite-related structure

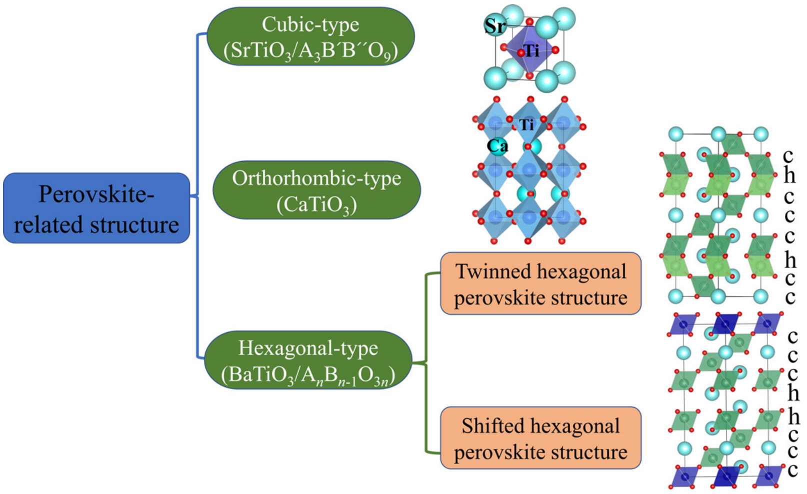

**Fig. 17** Classification and the representative structure of perovskite-related structure.

contained cubic-type, orthorhombic-type, and hexagonal-type structures. For hexagonal-type structure, the twinned hexagonal structure means the closely packed $AO_3$ layers were stacked in the order of $(ccch)_2$, while the shifted hexagonal structure corresponds to ccchhccc order. The typical representative of twinned structure is $Ba_8CoTa_6O_{24}$ and the shifted structure is $Ba_8CoNb_6O_{24}$ with eight-layer hexagonal perovskite structure [332]. The pseudo phase diagram of $ABO_3$ and complex $ABO_3$ type is provided in Fig. 18. From cubic and orthorhombic to hexagonal perovskite structure, researchers have proposed that tolerance factor, distortion of octahedron, and temperature of phase transition determined the variation of $\tau_f$ value, and the ordered/disordered cations were primarily related to quality factor.

### 2.5.1 *$ABO_3$ formula*

This section contains the ceramics with a general

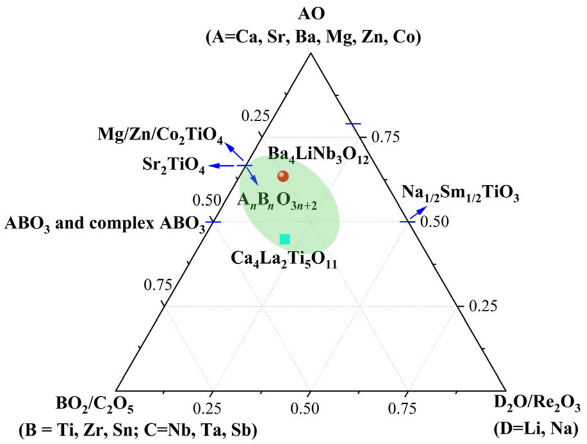

**Fig. 18** Pseudo phase diagram of perovskite related structure.

formula of $ABO_3$ and their related structure or system. Perovskite family is entirely studied because of their pyro and piezo electricity, linear and non-linear electric–optic properties, and superconducting properties. A serials of investigation of $CaTiO_3$ with $MgTiO_3$-based [333–339], $LaAlO_3$-based [340–347], $LaGaO_3$ [348], $Re(Zn_{1/2}Ti_{1/2})O_3$ [349], $(Mg_{1-x}Zn_x)_{1.8}Ti_{1.1}O_4$ [350], $BiSbO_4$ [351], $Nd(Mg_{0.4}Zn_{0.1}Sn_{0.5})O_3$ [352], $Sm_{0.9}Nd_{0.1}AlO_3$ [353], $Ca(Mg_{1/3}Nb_{2/3})O_3$ [354], $(Li_{0.5}La_{0.5})TiO_3$ [355], $Li_{0.5}Nd_{0.5}TiO_3$ [356], $Li_{0.5}Sm_{0.5}TiO_3$ [357], $Mg_{0.95}Co_{0.05}TiO_3$ [335], $(Sm,Nd)AlO_3$ [358–364], $Zn_2SnO_4$ [365], $Li_3NbO_4$ [248], $Ca(Mg_{1/3}Nb_{2/3})O_3$ [366], $Sm_{0.9}Nd_{0.1}AlO_3$ [367], $Na_{0.5}Nd_{0.5}TiO_3$ [368], $Nd(Mg_{1/2}Ti_{1/2})O_3$ [369,370], $Bi_{0.5}Na_{0.5}TiO_3$ [371], $3CaO–Re_2O_3–2WO_3$ system [372], $Ba_{0.6}Sr_{0.4}La_4Ti_4O_{15}$ [373], $La_2MgGeO_6$ [374], $(Ca_{0.8}Sr_{0.2})(Sn_xTi_{1-x})O_3$ [375], $Ca_{0.7}Ti_{0.7}La_{0.3}(Al_{0.3-x}Ga_x)O_3$ [376], $Ca_{(1-x)}Nd_{2x/3}TiO_3$ [377], $Ca_{0.6}La_{0.2667}TiO_3$ [378], $Ca_{0.6}La_{0.267}Ti_{1-x}(Nb_{0.5}Ga_{0.5})_xO_3$ [379], $Ca_{0.6}Nd_{0.8/3}TiO_3$–$(Li_{0.5}Nd_{0.5})TiO_3$ [380], $Ca_{0.4-x}Mg_xSm_{0.4}TiO_3$ [381], $CaTi_{1-x}(Nb_{0.5}Ga_{0.5})_xO_3$ [382], $Ca_{0.66}Ti_{0.66}Sm_{0.34}Al_{0.34}O_3$ [383], $Ca_{0.66}Ti_{0.66}Nd_{0.34}Al_{0.34}O_3$ [384], $CaTi_{1-x}(Al_{0.5}Nb_{0.5})_xO_3$ [385,386], $Ca_{0.6}La_{0.8/3}(Sn_xTi_{1-x})O_3$ [387], $Ca_{(1-3x/2)}Ce_xTiO_3$ [388], $Ca_{0.35}Li_{0.25}Nd_{0.35}Ti_{1-x}(Zn_{1/3}Ta_{2/3})_xO_3$ [389], $Ca_{0.61}Nd_{0.26}Ti_{1-x}Cr_xO_3$ [390], $CaTi_{1-x}(Mg_{1/2}W_{1/2})_xO_3$ [391], and $Ca(Hf_xTi_{1-x})O_3$ [392] ceramics have been reported thoroughly. Different thermally treated methods to minimize the dielectric loss for $CaTiO_3$ were proposed by Hu *et al.* [393]. The dielectric constant values saturated at 7.7–8.5 of $Ca_{0.8}Sr_{0.2}SnO_3$ ceramics in the sintering temperature range of 1450–1540 ℃ [394]. $0.4Nd_{2.94/3}Ba_{0.03}(Mg_{0.5}Sn_{0.5})O_3$–$0.6Ca_{0.8}Sr_{0.2}TiO_3$ ceramics modified the $\tau_f$ value to –7 ppm/℃ when sintered at 1600 ℃ [395]. Although numbers of investigations about optimizing the properties of $CaTiO_3$ ceramics have been reported, the vibrational characteristic of $CaTiO_3$ was verified by Shi *et al.* [396] in 2020.

For solid solution of $Ba[Ti_{0.4}Ga_{0.3}Nb_{0.3(1-x)}Sb_{0.3x}]O_3$, a near zero $\tau_f$ value of –1.1 ppm/℃ was obtained with $x$ = 0.5 [397], while a $\tau_f$ value of 8.2 ppm/℃ was achieved for $(Sr_{0.2}Ga_{0.488}Nd_{0.208})Ti_{1-x}Ga_{4x/3}O_3$ with $x$ = 0.5 [398]. A dramatical decrease of $\tau_f$ value from 1171 to –82 ppm/℃ was obtained for $Sr(Zr_xTi_{1-x})O_3$ [399]. In the chemical formula of $SrO(Sr_{1-x}Ba_xTiO_3)_n$ ($x$ = 0, 0.5; $n$ = 1–4), it is demonstrated that samples with $n$ = 1, 2 had no dielectric non-linear behavior in the temperature range of (–165)–50 ℃, while the tunability increased with $n$ increasing [400]. Two second phases containing $BaWO_4$ and $Ba_2Ti_5O_{12}$ were observed in $Ba_{0.5}Sr_{0.5}Ti1_{-3y/2}W_yO_3$ system with $y \geqslant 0.02$ [401], and $BaTiSiO_5$ phase was indexed in $Ba_{0.4}Sr_{0.6}Ti_{1-y}Si_yO_3$ [402]. The dielectric constant can be adjusted apparently in the $Ba_{0.4}Sr_{0.6}TiO_3$–$BaMoO_4$ and $Ba_{0.5}Sr_{0.5}TiO_3$–$AMoO_4$ (A = Ba, Sr) composite ceramics, where only cubic perovskite structure and scheelite structure were detected [403,404]. However, the $BaMoO_4$ was observed when $MgMoO_4$ added into $Ba_{0.5}Sr_{0.5}TiO_3$ [405]. Adding $Zr_{0.8}Sn_{0.2}TiO_4$ into $Ba_{0.4}Sr_{0.6}TiO_3$, the dielectric constant and dielectric loss increased with the increase of the content of $Zr_{0.8}Sn_{0.2}TiO_4$ [406]. Adding Fe power in $Ba_{0.4}Sr_{0.6}TiO_3$ ceramics indicated that the appearance of $Fe^{2+}$ and $Fe^{3+}$ would decrease the O vacancy concentrations and enhance the microwave dielectric properties [407]. In the ternary system of $Ba_{0.5}Sr_{0.5}TiO_3$–$MgO$–$Mg_2TiO_4$ [408], $Ba_{0.5}Sr_{0.5}TiO_3$–$MgO$–$Mg_2SiO_4$ [409], and $(1-x-y)BaTiO_3$–$xCr_2Ti_3O_9$–$yBi_2O_3$ [410], the dielectric constant reduced with more $Mg_2TiO_4$ and $Mg_2SiO_4$, while solid solution was observed with $Cr^{3+}$ and $Bi^{3+}$ into $BaTiO_3$. Co-doping ZnO, $Al_2O_3$, and MgO on the $Ba_{0.66}Sr_{0.4}TiO_3$ would generate $Mg(Zn)Al_2O_4$ as the secondary phase [411]. The lattice vibrations of $Ba_{0.4}Sr_{0.6}TiO_3$ ceramics were systematically investigated by Jiang *et al.* [412] after Mn substituted for Sr. Zr and Sn doped into $Ba_{0.1}Mg_{0.9}TiO_3$ ceramic would bring about $Ba_2Ti_9O_{20}$ and $BaTi_5O_{11}$ [413]. Similar to $CaTiO_3$, the effect of $LnAlO_3$ (Ln = Sm, Nd) on $BaTiO_3$-based ceramics was systematically studied by Liu *et al.* [414] and Xie *et al.* [415]. Solid solution of $Ba_xMg_{1-x}Ti_{0.95}Sn_{0.05}O_3$ [416] and local 1:1 ordering in B-site of $Sr(Ga_{0.5}Nb_{0.5})_{1-x}Ti_xO_3$ was verified by TEM and Raman spectroscopy, and the decline of quality factor stemmed from the anharmonicity by Ti substitution [417]. $0.2SrTiO_3$–$0.8Ca_{0.61}Nd_{0.26}Ti_{1-x}Al_{4x/3}O_3$ ceramics also reached a near zero $\tau_f$ value with $x$ = 0.5 [418]. $(1-x)Mg(Ti_{0.95}Sn_{0.05})O_3$–$xBaTiO_3$ compounds experienced a phase transition of tetragonal-structure $BaTiO_3$, monoclinic-structure $Ba_4Ti_{11}O_{26}$, and triclinic-structure $Ba_2Ti_9O_{20}$ [419]. Likewise, $Sr_{(1-1.5x)}Ce_xTiO_3$ ($x$ = 0.1–0.67) ceramics changed from cubic, tetragonal, to orthorhombic, and the dielectric behaviors were dominated by oxygen vacancies and defect dipoles [420]. Tian *et al.* [421,422] reported that $(Co_{0.5}W_{0.5})^{4+}$ and $(Zn_{0.5}W_{0.5})^{4+}$ occupying the Ti-site in $BaTiO_3$ would render the $\tau_f$ value change from positive to negative. $BaWO_4$ phase appeared in $Ba_{1-x}Sr_x(Mg_{0.5}W_{0.5})O_3$ ceramics and $Ba_2Mg_{0.95}Zn_{0.05}WO_6$, and the grain size distributed in a narrow range around 0.8 μm [423,424]. A $\tau_f$ value of –2.4 ppm/℃ was achieved for B-site deficient $Ba(Mg_{(1-x)/2}Y_{x/3}\square_{x/6}W_{1/2})O_3$ [425]. In the non-stoichiometric system of $(Sr_{0.4}Ce_{0.4})_{1-x}Nd_xTi_{0.8}Mg_{0.2}O_3$, solid solution was obtained when $x \leqslant 0.2$, while the satisfied properties were $\varepsilon_r \approx 53$, $Q\times f$ value ≈ 26,700 GHz, and $\tau_f \approx +2.8$ ppm/℃ with $x$ = 0.4 [426]. Meanwhile, compositional dependence of microwave dielectric properties of doped $SrTiO_3$ sintered in air is presented as Fig. 19. It was demonstrated that $SrTiO_3$ added into $ZnAl_2O_4$–$3Zn_2SiO_4$–$2SiO_2$ could reduce the sintering temperature from 1320 to 1180–1200 ℃ [427].

With the same general formula of $ABO_3$, the investigations of $NdGaO_3$, $NdNbO_3$, and $AgTa/NbO_3$ are listed adjacently to $CaTiO_3$ and $SrTiO_3$. Phase composition was identified for $NdGaO_3$–$Bi_{0.5}Na_{0.5}TiO_3$ system, and new temperature-stable ceramics with $0.4NdGaO_3$–$0.6Bi_{0.5}Na_{0.5}TiO_3$ was obtained [428]. Order–disorder transformation of A-site-deficient perovskites plays a significant role in conductivity of materials. The investigation of crystal structure and dielectric properties of the $Nd_{(1-x)/3}M_xNbO_3$ (M = Li, Ag; $0 \leqslant x \leqslant 0.2$) suggested that the dielectric loss majored by the lithium or silver ionic conduction at low frequencies [429]. Solid solution of $AgNb/TaO_3$-based ceramics was then studied extensively [430,431]. Temperature-stable MWDCs with the formula of $(La,Nd)_{2/3}TiO_3$ were studied by Saleem *et al.* [432].

$MgTiO_3$ also belongs to the general formula of $ABO_3$. The substitution for $MgTiO_3$ such as Ni, Zn, Co, and Mn for Mg has been investigated systematically [433–436], where $(Mg_{1-x}Co_x)TiO_3$ ceramics were crystalized as ilmenite structure when $x \leqslant 0.5$, and the

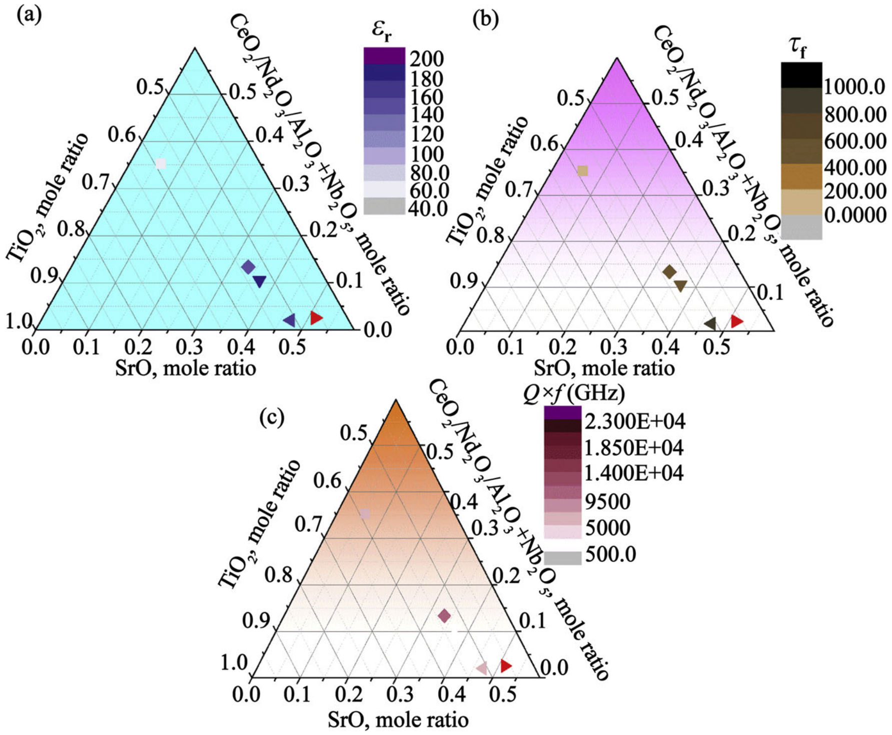

**Fig. 19** Compositional dependence of microwave dielectric characteristics of $Nd_2O_3$, $CeO_2$, $Al_2O_3$, and $Nb_2O_3$ doped $SrTiO_3$ compound sintered in air, closed pipe, and nitrogen atmosphere with (a) relative permittivity ($\varepsilon_r$), (b) TCF ($\tau_f$), and (c) $Q\times f$ value, respectively. Reproduced with permission from Ref. [426], © The Chinese Ceramic Society 2020.

secondary phase was detected with more doping cations [437]. $(Zn_{1-x}Mg_x)TiO_3$ was prepared and demonstrated that the dielectric constant and loss decreased with Mg increase [438]. For Sn doped into Ti site in $MgTiO_3$, in the range of $x$ = 0.05–0.07, the ceramics exhibited excellent microwave dielectric properties of $\varepsilon_r \approx$ 16.8–17.1, $Q\times f$ value ≈ 298,000–312,000 GHz, and $\tau_f \approx$ (–53)–(–50) ppm/℃ [439]. $Mg_{0.95}Co_{0.05}TiO_3$ ceramics possessed properties as $\varepsilon_r \approx$ 17.03, $Q\times f$ value ≈ 170 THz, and $\tau_f \approx$ –40 ppm/℃ when prepared by Semi Alkoxide precursor method [440]. Gong *et al.* [441] obtained $Mg(Sn_{0.05}Ti_{0.95})O_3$ ceramics with microwave dielectric properties $\varepsilon_r \approx$17.6, $Q\times f$ value ≈ 328,543 GHz, and $\tau_f \approx$ –42 ppm/℃, and Jia *et al.* [442] proposed that $Mg(Ti_{1-x}Nb_x)O_3$ showed microwave dielectric properties: $\varepsilon_r \approx$ 18.12, $Q\times f$ value ≈ 163,618 GHz, and $\tau_f \approx$ –40.1 ppm/℃. Through sol–gel process, the quality factor of geikielite-type $MgTiO_3$ saturated when the ceramics sintered at 1200 ℃ [443]. After adding $B_2O_3$ into $MgTiO_3$, the composite ceramics could be densified at 1100 ℃ [444]. Investigation of introduction $SrTiO_3$ into $Mg(Zr_{0.05}Ti_{0.95})O_3$ ceramics suggested that a close zero $\tau_f$ value could achieve at $0.96Mg(Zr_{0.05}Ti_{0.95})O_3$–$0.04SrTiO_3$ [445,446]. In the study of a designed composition of $MgTiO_3$ (Mg/Ti = 1, 1.02, 1.04, 1.05, 1.07), the generation of $MgTi_2O_5$ which derived from Mg/Ti = 1 was restrained, and then pure phase of $MgTiO_3$ was obtained when Mg/Ti = 1.02 [447]. $(Co_{1-x}Zn_x)TiO_3$ sintered at 1350 ℃ possessed $\varepsilon_r \approx$ 20, $Q\times f$ value ≈ 107,000GHz, and $\tau_f \approx$ –60 ppm/℃ with $x$ = 0.05 [448]. The choice of raw material of MgO and $Mg(OH)_2$ had a major influence on the phase formation and dielectric loss for $0.97MgTiO_3$–$0.03SrTiO_3$ [449]. In the system of $(1-x)MgTiO_{3-x}Mg_{2.05}SiO_{4.05}$–$0.06CaTiO_3$, $\tau_f \approx$ 1.45 ppm/℃ was obtained with $x$ = 0.2 [450]. $ZnTiO_3$-type phase, $Zn_2TiO_4$-type, and $TiO_2$ phase were co-existed in $(Zn_{0.3}Co_{0.7})Ti_{1-x}Sn_xO_3$, and the satisfied microwave dielectric properties were $\varepsilon_r \approx$ 24, $Q\times f$ value ≈ 66,700GHz, and $\tau_f \approx$ –5.43 ppm/℃ with $x$ = 0.02 [451]. It was interesting that $MgTiO_3$ and $Mg_2TiO_4$ were the main phases in $Mg_{n+1}Ti_nO_{3n+1}$ ($n$ = 2, 3, 4, 5, 6, and 7), and the $Mg_2TiO_4$ was effectively inhibited with $n$ increasing [452]. New cofired tri-layer ceramic architecture of $MgTiO_3/TiO_2/MgTiO_3$ was designed to realize the temperature-stable and ultrahigh-$Q$ ceramics, where the property comparison of MgO–$TiO_2$ system (Fig. 20) indicated that this new strategy was effective for developing high-performance dielectric resonators [4]. 2 wt% $B_2O_3$ as an addictive could effectively reduce the sintering temperature from

1275 to 1175 ℃ in $0.9625MgTiO_3$–$0.0375Ca_{0.5}Sr_{0.5}TiO_3$ [453]. $Mg_2TiO_4$-related and $Mg_6Ti_5O_{16}$-based ceramics in MgO–$TiO_2$ system were also reported. $Mg_6Ti_5O_{16}$-based MWDCs were systematically investigated by Yu *et al.* [454], where the $\tau_f$ value could be adjusted to –3 ppm/℃ by $Ca^{2+}$ substitution. To explore the application for mobile communication, $Nb^{5+}$ ion was added into $Mg_2SnO_4$ to improve the quality factor [455]. By mechanical synthesis method, the value of quality factor was sensitive to the initial particle size and microstructure of $Mg_2TiO_4$ [456]. Meanwhile, the oxygen vacancies and average sizes were highly influenced on the dielectric loss of adding $La_2O_3$, $V_2O_5$, and $CeO_2$ into $Mg_2TiO_4$ [457,458]. A maximum quality factor value of 210,700 GHz was obtained in $(Mg_{1-x}Zn_x)_{1.8}Ti_{1.1}O_4$ with $x$ = 0.06 [459]. $[(Mg_{0.5}Zn_{0.5})_{0.95}Co_{0.05}]_2TiO_4$ was demonstrated as the optimal composition in the solid solution of (Mg, $Zn)_2TiO_4$–$Co_2TiO_4$ with $Q\times f$ value ≈ 2100,000 GHz [460]. The average particle size of pure $Mg_2TiO_4$ nano-powders was reduced to 163 nm via high energy ball milling method, and the excellent properties were $\varepsilon_r$ ≈ 13.9, $Q\times f$ value ≈ 98,600 GHz, and $\tau_f$ ≈ –50.9 ppm/℃ [461]. Similarly to the formula of $Mg_2TiO_4$, spinel-based $CoZnTiO_4$ ceramics were obtained after sintered at 1200 ℃, and the properties were majored by the relative density and grain size [462]. Solid solution of $Mg_2(Ti_{1-x}Sn_x)O_4$ [463] and $ZnNiTiO_4$/$ZnNiTi_{1-x}Sn_xO_4$ [464,465] was also reported. Until now, the intrinsic dielectric behavior of $Mg_2TiO_4$ based on P–V–L theory and infrared spectra was presented by Li *et al.* [466], where the Ti(1)–O bond plays a primary role in dielectric loss. Meanwhile, $Mg_2Ti_{1-x}Ga_{4x/3}O_4$ would reach a $Q\times f$ value ≈ 205,416 GHz [467].

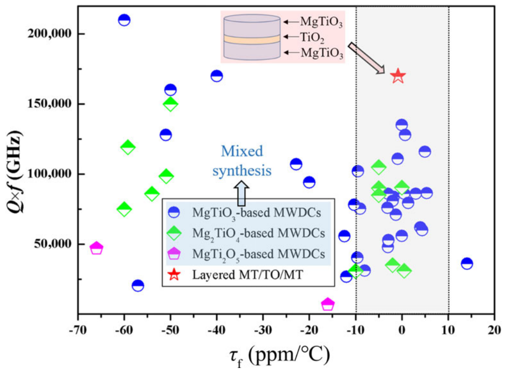

**Fig. 20** Summary of $Q\times f$ value versus $\tau_f$ plot for MgO–$TiO_2$ system MWDCs. Reproduced with permission from Ref. [4], © Elsevier Ltd and Techna Group S.r.l. 2018.

Furthermore, there are some compounds in the formula of $Na_{0.5}Ln_{0.5}TiO_3$ (Ln = Sm, Nd). Fang *et al.* [468] and Zhou *et al.* [469] reported a serials of substitution, such as $Na_{0.5}Nd_{0.2}Sm_{0.3}Ti_{1-x}Sn_xO_3$, $Na_{0.5}Nd_{0.5}Ti_{1-x}Sn_xO_3$, $Na_{0.5}Nd_{0.2}Sm_{0.3}Ti_{1-x}Zr_xO_3$ [470], and $Na_{1/2}Sm_{1/2}Ti_{1-x}(Cr_{1/2}Nb_{1/2})_xO_3$ [471]. Near zero $\tau_f$ value appeared in $Li_{0.5}Sm_{0.5}TiO_3$–$Na_{0.5}Sm_{0.5}TiO_3$ [472].

### 2.5.2 $A_2B'B''O_6$ formula

Due to the flexibility and adjustability of the crystal structure of perovskite, the investigation of complex perovskite with various cations occupying Ti site gradually emerged. The structural studies of $A_2B'B''O_6$ (A = Ba, Sr, Ca; B′ = lanthanide, Mg, Cr, Bi; B″ = Nb, Ta, Sb, W) indicated that phase transitions were ascribed to the tilting of $B'O_6$/$B''O_6$. In the $Ba_{2-2x}Sr_{2x}SmSbO_6$ system, phase transitions of $Fm\overline{3}m$, $I_2/m$, and $P2_1/n$ were observed and the $\tau_f$ value shifted from +25 to –50 ppm/℃ [473]. Effect of non-stoichiometry $Ba_{1+x}(MgW)_{1/2}O_3$, $Ba(Mg_{1+y}W)_{1/2}O_3$, and $Ba(MgW_{1+z})_{1/2}O_3$ and the sintering temperature on microwave dielectric properties was systematically investigated by Wu and Bian [474] and Chen *et al.* [475], respectively. A zero $\tau_f$ value ceramic was obtained in $Ba_2Mg_{1-x}Ca_xWO_6$ system with $x$ = 0.1 [474]. First-principles calculation of assignment for vibrational spectra of $Ba(Mg_{1/2}W_{1/2})O_3$ MWDCs is shown in Fig. 21 [476], which proposed that $F_{1u}(2)$ modes originated from Mg–$O_6$ vibrations had the largest contribution to the dielectric properties. The investigation of microwave dielectric properties of giant permittivity ceramics with a $A_2B'B''O_6$ formula $(Ba(Fe_{1/2}Nb_{1/2})O_3$ and $Sr(Fe_{1/2}Nb_{1/2})O_3)$ indicated that the permittivity was independent of frequency [477].

$Ln(B_{0.5}C_{0.5})O_3$ (Ln = La, Sm, Nd; B = Mg, Zn; C = Ti, Sn) ceramics belonging to the general formula of $A_2B'B''O_6$ have been reported as low dielectric loss materials with an adjustable temperature coefficient of resonant frequency. Among them, minor amount of low-melt point oxide of $Bi_2O_3$ and $B_2O_3$ was usually used to enhance the sintering densification of $Sm(Mg_{0.5}Ti_{0.5})O_3$ [478,479], CuO was added into $La_{2.98/3}Sr_{0.01}(Mg_{0.5}Sn_{0.5})O_3$ to enhance the densification [480], and $V_2O_5$ was valid for reducing the sintering temperature of $Nd(Zn_{1/2}Ti_{1/2})O_3$ [481]. Solid solution of $Nd_{(1-x)}Sm_x(Mg_{0.5}Sn_{0.5})O_3$ [482], $Nd(Mg_{0.5-x}Co_xSn_{0.5})O_3$ [483], $Nd_{(1-2x/3)}Ca_x(Mg_{0.5}Sn_{0.5})O_3$ [484], $Nd_{(1-2x/3)}Sr_x(Mg_{0.5}Sn_{0.5})O_3$ [485], $Nd_{(1-2x/3)}Ba_x(Mg_{0.5}Sn_{0.5})O_3$ [486],

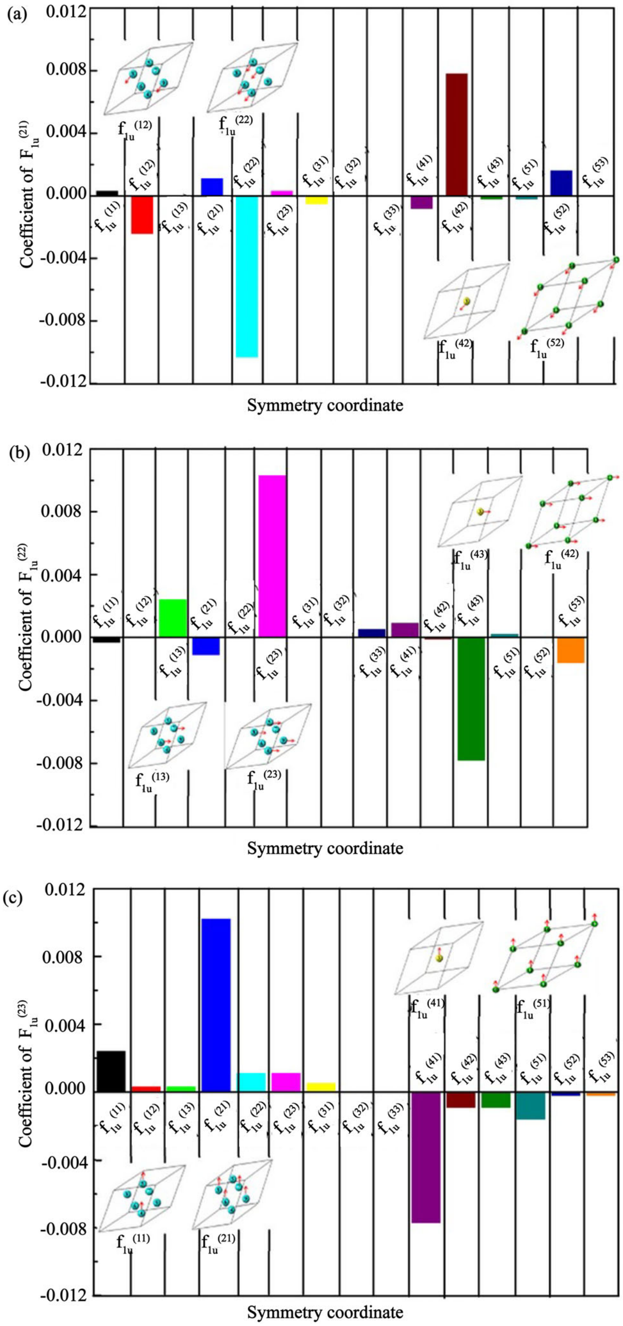

**Fig. 21** Combinations of the symmetry coordinates (normalized) for IR-active $F_{1u}(2)$ modes. Reproduced with permission from Ref. [476], © The American Ceramic Society 2013.

$Nd(Mg_{0.5-x}Ni_xSn_{0.5})O_3$ [487], $Nd(Mg_{0.5-x}Zn_xSn_{0.5})O_3$ [488], $Nd(Mg_{0.5}Sn_{0.5-x}Ti_x)O_3$ [489], $Nd(Mg_{0.5-x}Ca_xSn_{0.5})O_3$ [490], Nd $[(Zn_{1-x}Co_x)_{0.5}Ti_{0.5}]O_3$ [491], $Nd(Zn_{0.5+x}Ti_{0.5})O_3$ [492], $Nd_{(1+x)}(Mg_{1/2}Sn_{1/2})O_3$ [493], $Nd(Mg_{0.5}Sn_{0.5(1+x)})O_3$ [494], $La_{1-x}B_x(Mg_{0.5}Sn_{0.5})O_3$ [495], $La_{(1-x)}Sm_x(Mg_{0.5}Sn_{0.5})O_3$ [495], $La_{0.97}Sm_{0.03}(Mg_{0.5}Sn_{0.5})O_3$–$Ca_{0.8}Sr_{0.2}TiO_3$ [395], $La_{1-x}Yb_x(Mg_{0.5}Sn_{0.5})O_3$ [496], $La(Mg_{0.5-x}Ba_xSn_{0.5})O_3$ [497], $Pr_{0.22}Y_{0.78}TiTaO_6$ [498], $La(Mg_{0.4}Sr_{0.1}Sn_{0.5})O_3$ $Nd(Co_{0.5}Ti_{0.5})O_3$–$Ca_{0.8}Sr_{0.2}TiO_3$ [499,500], $La(Mg_{0.5-x}Sr_xSn_{0.5})O_3$ [501], $Ca_{0.6}La_{0.267}TiO_3$–$Ca(Sm_{0.5}Nb_{0.5})O_3$ [502], and $La[Al_{1-x}(Mg_{0.5}Ti_{0.5})_x]O_3$ [503] was investigated based on sintering behavior and microstructure. Not only the investigations reported the microwave dielectric properties, but also the structure–property relationship containing intrinsic loss, vibrational modes, and chemical bond characteristics of $Y_2MgTiO_6$ was studied in detail, and the schematic representation of vibrational modes of Y site was presented in Fig. 22 [504].

### 2.5.3 $A(B'_{1/3}B''_{2/3})O_3$ formula

$A(B'_{1/3}B''_{2/3})O_3$ (A = Ba, Ca; B′ = Mg, Zn; B″ = Nb, Ta) ceramics have been commercially used due to their excellent $Q\times f$ value, and the near-zero $\tau_f$ value. The order structures of $Ca_{1-0.3x}La_{0.2x}[(Mg_{1/3}Ta_{2/3})_{1-x}Ti_x]O_3$-based, $Ba(Mg_{1/3}Nb_{2/3})O_3$-based, $Ba(Zn_{1/3}Ta_{2/3})O_3$-based and $Ba_3CaNb_2O_9$ ceramics were investigated by TEM and the vibrational spectra to explain the cation ordering [505–525]. Meanwhile, superstructure reflections were obviously recorded in this system, such as $Ba(Zn_{1/3}Ta_{2/3})O_3$ doped with $Nb_2O_5$, $MnO_2$, and $V_2O_3$ [526,527]. The wavelength of 1:2 ordered superlattice modulation was about 0.71 nm, while that of disordered superlattice modulation was 0.41 nm of $Ba((Co_{0.6-x/2}Zn_{0.4-x/2}Mg_x)_{1/3}Nb_{2/3})O_3$, shown as Fig. 23 [511]. Adding $MnO_2$ into $Ba(Co_{1/3}Nb_{2/3})O_3$ would enhance the grain growth and restrain the evaporation of CoO [527]. Meanwhile, the influence of B″-site non-stoichiometry of $Ba(Co_{0.56}Y_{0.04}Zn_{0.35})_{1/3}Nb_{2/3+x}$ on properties was reported by Tang *et al.* [528], where $Ba_5Nb_4O_{15}$ as a secondary phase was recorded. Simulation is carried out for $Ba(Zn_{1/3}Ta_{2/3})O_3$ for the design of linear metal taper [529]. Peng *et al.* [530] reported that addition of $La_2O_3$ into $Ba(Mg_{1/3}Ta_{2/3})O_3$, $Ba_{1-x}Ca_x(Mg_{1/3}Ta_{2/3})O_3$, and $Ba[Mg_{1-x}Zn_x]_{1/3}Ta_{2/3}O_3$ led to the appearance of $Ba_{0.5}TaO_3$, and $\tau_f$ value reached to near zero [531,532]. The optimal properties of $Ba[Mg_{(1-x)/3}Sn_xTa_{2(1-x)/3}]O_3$ exhibited as $\varepsilon_r \approx 24.1$, $Q\times f$ value ≈ 138,500 GHz, and $\tau_f \approx +0.2$ ppm/℃ [533]. The variation of $\tau_f$ values for 1:1 and 1:2 complex perovskites was clarified to be mainly relevant with tolerance factors, which are summarized in Fig. 24 [524]. It has been verified that samples with non-stoichiometric $Mg^{2+}$ and $Ta^{5+}$ in $Ba(Mg_{1/3}Ta_{2/3})O_3$ exhibited a wide temperature stability [525,534], and the correlations between $Q\times f$ versus $\varepsilon_r$ and $\tau_f$ versus $\varepsilon_r$ of high-$Q$ (⩾ 100,000 GHz) MWDCs are presented in Fig. 25.

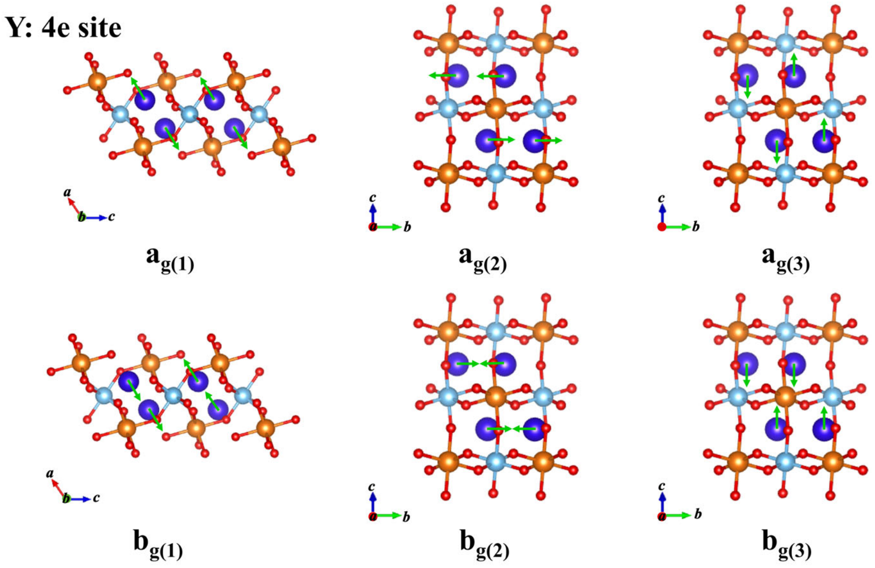

**Fig. 22** Schematic representations of the vibrational modes of $Y_2MgTiO_6$ system (Y at 4e site). Reproduced with permission from Ref. [504], © The American Ceramic Society 2019.

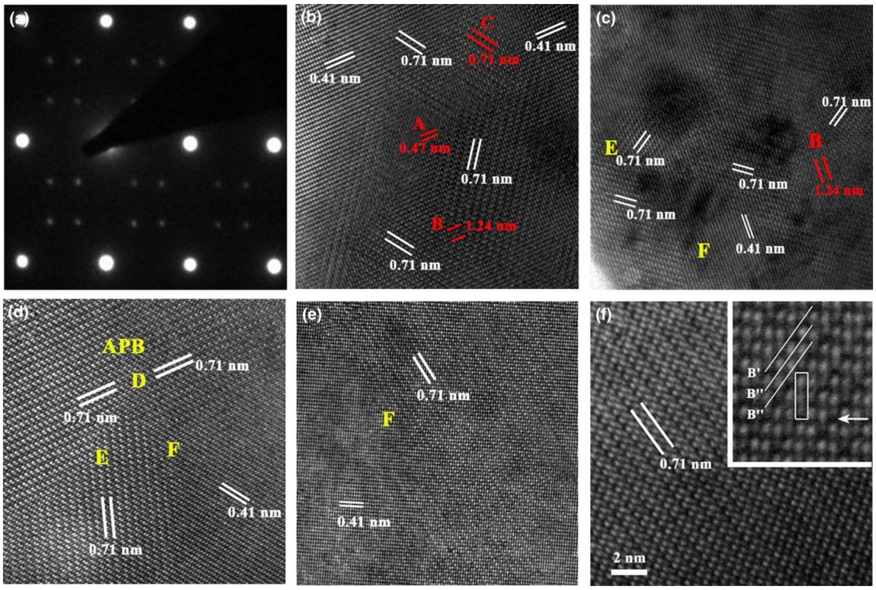

**Fig. 23** SAED pattern with zone axis $[1\bar{1}0]_c$ and the corresponding HRTEM images for $Ba((Co_{0.6-x/2}Zn_{0.4-x/2}Mg_x)_{1/3}Nb_{2/3})O_3$ ceramics: (a) $x = 0.1$; (b) $x = 0$; (c) $x = 0.1$; (d) $x = 0.2$; (e) $x = 0.3$; (f) HRTEM image of an ordered area in high magnification. Reproduced with permission from Ref. [511], © The American Ceramic Society 2013.

### 2.5.4 *$A_nB_nO_{3n+2}$ formula*

Perovskite-related oxides of series $A_nB_nO_{3n+2} = ABO_x$ ($x = 3+2/n$) (A = Ca, Sr, or La and B = Ti or Nb) with $n$ = 4, 4.33, 4.5, 5, 6, and 7 have been a focus owing to their electronic and dielectric properties. The crystal type and the physical properties rely on the value of $n$, which descripts the number of octahedral layers in the slabs [535]. Besides $Ca_5Nb_5O_{17}$, the $A_nB_nO_{3n+2}$ phases appeared in the binary system of $La_2Ti_2O_7$–$CaTiO_3$, $Nd_2Ti_2O_7$–$CaTiO_3$, and $Ca_2Nb_2O_7$–$CaTiO_3$. Joseph *et al.* [536] reported the microwave dielectric properties of $Ca_5A_4TiO_{17}$ (A = Nb, Ta) as $\varepsilon_r \approx 44.9$, $Q \times f$ value ≈ 17,600 GHz, and $\tau_f \approx -112.9$ ppm/℃ for $Ca_5Nb_4TiO_{17}$; $\varepsilon_r \approx 40.1$, $Q \times f$ value ≈ 16,500 GHz, and $\tau_f \approx -53.6$ ppm/℃ for $Ca_5Ta_4TiO_{17}$. The solid solution of $SrLa_{4-x}Sm_xTi_5O_{17}$ ($0 \leqslant x \leqslant 4$) and $Sr_{1-x}Ca_xLa_{4-x}Ti_5O_{17}$ ($0 \leqslant x \leqslant 1$) would lower the $\tau_f$ to zero with a dielectric constant of near 53 [537,538], while $\tau_f$ declined to +70 ppm/℃ by Zr substituted for Ti of

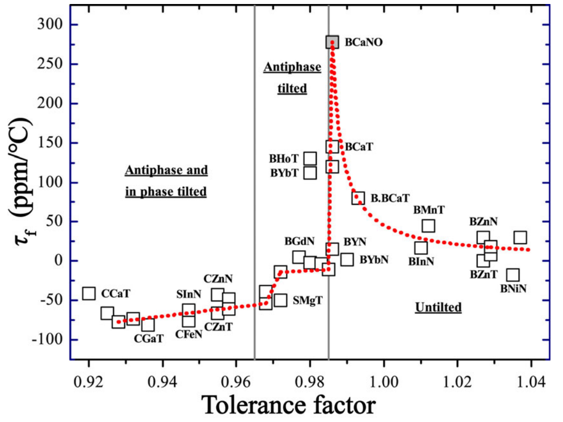

**Fig. 24** Temperature coefficient of resonant frequency ($\tau_f$) versus tolerance factor ($t$) for A(B′,B″)$O_3$ (A = Ca, Sr, Ba; B′ = Mg, Ca, Mn, Fe, Ni, Zn, Ga, In, Y, Gd, Tb, Dy, Ho, Er, Yb; B″ = Nb, Ta) 1:1 and 1:2 complex perovskites. Reproduced with permission from Ref. [524], © Elsevier Ltd and Techna Group S.r.l. 2016.

$SrLa_4Ti_5O_{17}$ [539]. The intermediate of two end member phases of $CaLa_4Ti_5O_{17}$ and $Ca_5Nb_4TiO_{17}$ showed that the $\varepsilon_r$ varied from 45 to 52, $Q\times f$ was in the range of 9870–5680 GHz, and $\tau_f$ value ranged between –38 and –126.4 ppm/℃ [540]. $La_3Ti_2TaO_{11}$ is an member of $n$ = 3 in this series, and the textured $La_3Ti_2TaO_{11}$ was fabricated by spark plasma sintering, showing that grain-orientation control was an effective way to tailor the properties of this ceramic [541]. $SrCa_4Nb_4TiO_{17}$ and $Ca_5Nb_4TiO_{17}$ sintered at their optimal temperature presented an elongated and plate-like grain [542]. From 0 to 4, the $\tau_f$ value shifted from –117 to 415 in $NaCa_{4-x}Sr_xNb_5O_{17}$ [543], while the $\tau_f$ value changed in the range of (–117)–473 ppm/℃ for $Na_{1-x}K_xCa_4Nb_5O_{17}$ [544].

### 2.5.5 $Ca_4La_2Ti_5O_{17}$

The dielectric properties of $Ca_4La_2Ti_5O_{17}$ were firstly reported by Rejini *et al.* [545], which were crystalized as perovskite structure and the XRD results were matched well based on the formula of $Ca_{0.706}La_{0.353}Ti_{0.882}O_3$. There are rare studies about this system, which just concentrated on the modification of $\tau_f$ value. For example, the dielectric constant declined from 71.86 to 35.23 in the solid solution of $Ca_4La_2Ti_{5-x}(Mg_{1/3}Nb_{2/3})_xO_{17}$ ($0 \leqslant x \leqslant 4$), and a near-zero $\tau_f$ value (1.62 ppm/℃) was achieved at $x$ = 3 [546]. Meanwhile, a near-zero $\tau_f$ value was measured for $0.4Ca_4La_2Ti_5O_{17}$–$0.6NdAlO_3$ ceramics [547] and $Mg_4La_2Ti_5O_{17}$ ceramics [548].

### 2.5.6 $A_nB_{n-1}O_{3n}$ formula

A series of $A_4B_3O_{12}$-type cation-deficient perovskite ceramics were consistent with the formula of $Sr_{4-m}La_mTi_{m-1}Ta_{4-m}O_{12}$ ($m$ = 1, 2, 3). $Sr_3LaNb_3O_{12}$ and $SrLa_3Ti_2NbO_{12}$ were firstly characterized by Fang *et al.* [549,550]. B-site deficient twinned perovskites such as $Ba_8Ti_3Nb_4O_{24}$, $Ba_8MTa_6O_{24}$ (M = Mg, Zn, Ni, Co, Cu), and $Ba_8Ga_{4-x}Ta_{4+0.6x}O_{24}$ are classified as $A_nB_{n-1}O_{3n}$

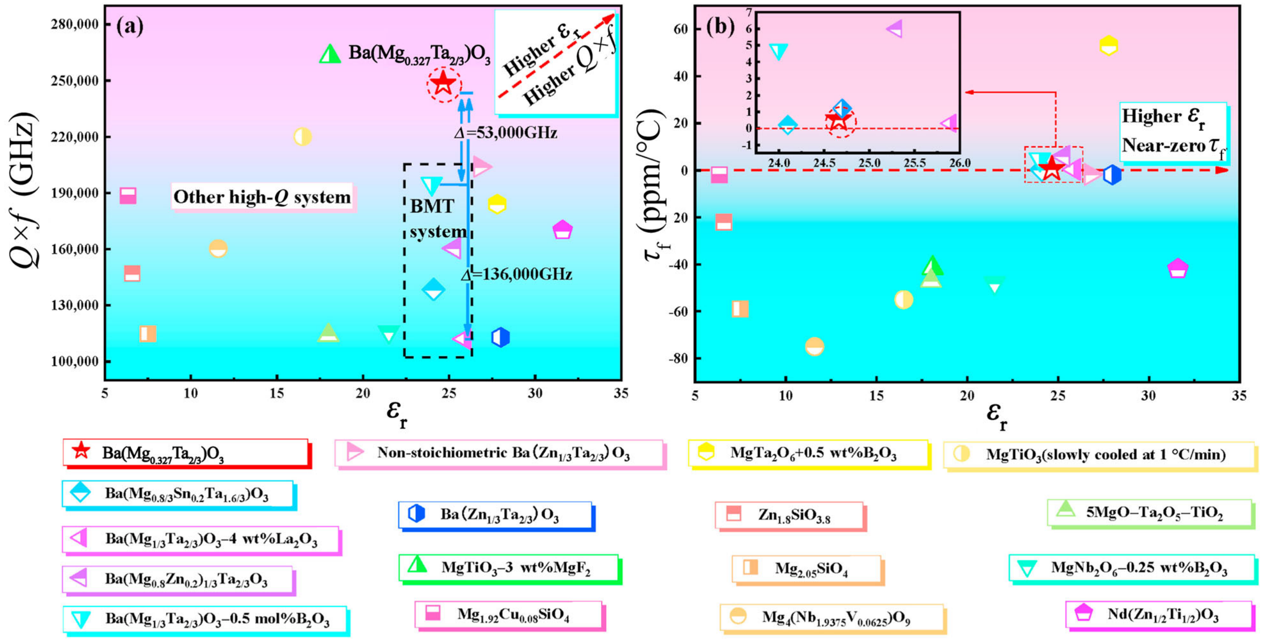

**Fig. 25** Correlation between microwave dielectric properties of high-$Q$ ($Q \times f \geqslant$ 100,000GHz) MWDCs: (a) $Q\times f$ versus $\varepsilon_r$; (b) $\tau_f$ versus $\varepsilon_r$. Reproduced with permission from Ref. [534], © Elsevier Ltd and Techna Group S.r.l. 2020.

hexagonal perovskites. $Ba_8ZnTa_6O_{24}$ is a secondary phase of $Ba(Zn_{1/3}Ta_{2/3})O_3$-based systems, and the dielectric properties in the range of 5 Hz–50 MHz of Sb substitution for Nb-site have been systematically studied by Suresh *et al.* [551,552] through spectroscopic methods. In the microwave frequency region, the $Q\times f$ value and $\tau_f$ values of $Ba_8(Mg_{1-x}Zn_x)Ta_6O_{24}$ ceramics decreased with the augment of $x$ [553]. Similarly, a single phase with hexagonal 8H perovskite structure of $Ba_8Ti_3Nb_{4-x}Sb_xO_{24}$ ceramics was prepared, and $\tau_f$ value declined from 110 to 2 ppm/℃ [554]. $BaWO_4$ was used to adjust the large $\tau_f$ value of 8H hexagonal perovskite $Ba_4LiNb_3O_{12}$, and the properties of $\varepsilon_r \approx 16.9$, $Q\times f$ value ≈ 75,500 GHz, and $\tau_f \approx +8.7$ ppm/℃ were obtained [555]. Phase transformation in the sequence of hexagonal, hexagonal along with cubic, and cubic was observed in $Ba_4LiNb_{3-x}Sb_xO_{12}$ and $Ba_4LiTa_{3-x}Sb_xO_{12}$ system. Especially, the optimal microwave dielectric properties were achieved for $Ba_4LiNb_2SbO_{12}$ with a zero $\tau_f$ [556,557]. $\tau_f$ value dropped from positive to negative in $Ba_3LiTa_{3-x}Sb_xTi_5O_{21}$ [558], and $Ba_3LiNb_{3-x}Sb_xTi_5O_{21}$ [559], while the $\tau_f$ value just reduced from 205 to 70 ppm/℃ for $Ba_3LiNb_{3-x}Ta_xTi_5O_{21}$ [560]. A-site deficient perovskite structure was well matched for $LiSmTa_4O_{12}$ ceramics with tetragonal perovskite structure (A-site deficient perovskite structure), and the optimal microwave dielectric properties were $\varepsilon_r \approx 59.60$, $Q\times f$ value ≈ 7760 GHz, and $\tau_f \approx +41.8$ ppm/℃ [561].

### 2.5.7 $Sr_{n+1}Ti_nO_{3n+1}$ ($n$ = 1, 2, 3 4, ∞) formula

Researchers paid their attention to Ruddlesden–Popper (R–P) structure until the dielectric properties of $CaReAlO_4$ (Re = Nd, Sm, Y) were reported. The general formula of R–P compounds was written as $(A,A')_{n+1}B_nO_{3n+1}$, where the structure was built by corner-sharing ($BO_6$) octahedral and interlayer of ((A,A′)O). $MLnAlO_4$ and $SrLn_2Al_2O_7$ (M = Ca, Sr; R = Y, Sm, Nd, La) belong to the R–P series with $n$ = 1 and 2, respectively. The crystal structures of $SrLaAlO_4$ and $SrLa_2Al_2O_7$ are presented in Fig. 26. Single crystals of $ABCO_4$ layered compounds with $K_2NiF_4$ structure were used as substrates for high-temperature superconductive thin films, while dielectric properties in this system were mainly investigated by Chen and his co-workers [562–574]. They contributed to analyze the relation between the intrinsic dielectric properties and crystal structure of $MRAlO_4$ (M = Ca, Sr; and R = Y, Sm, Nd, La). Combining the compression/dilation effects of different cation–oxygen bonds and the stability of crystal structure with vibrational spectrum, they emphasized that the drop of the quality factor was ascribed to the abnormal variations of axial bonds and the theoretical dielectric loss was obtained after fitted the infrared reflectivity spectra. With $(Zn_{0.5}Ti_{0.5})^{3+}$ substituted for $Al^{3+}$ of $SrLaAlO_4$, the best combination of microwave dielectric properties was $\varepsilon_r \approx 23.5$, $Q\times f$ value ≈ 102,000 GHz, and $\tau_f \approx -3.4$ ppm/℃ [572]. In the $SrLaAlO_4$–$Sr_2TiO_4$ system, some diffraction peaks shifted toward higher angles along with the variation of $x$, while some of them shifted toward lower angles, as shown in Fig. 27 [569]. This phenomenon was explained by the opposite change of $a$-axis and $c$-axis, where the octahedron elongated in the $ab$ plane with the shrinkage in the c direction. The tolerance factor ($t$) of perovskite layer was used to evaluate the stability of those compounds, and the relation of $t$ and $r(M^{2+})/r(Ln^{3+})$ was plotted in Fig. 28 [573]. $Sr_{0.6}Ca_{0.4}LaAlO_4$ with 10 wt% $TiO_2$ presented a near zero $\tau_f \approx -2.5$ ppm/℃ [575].

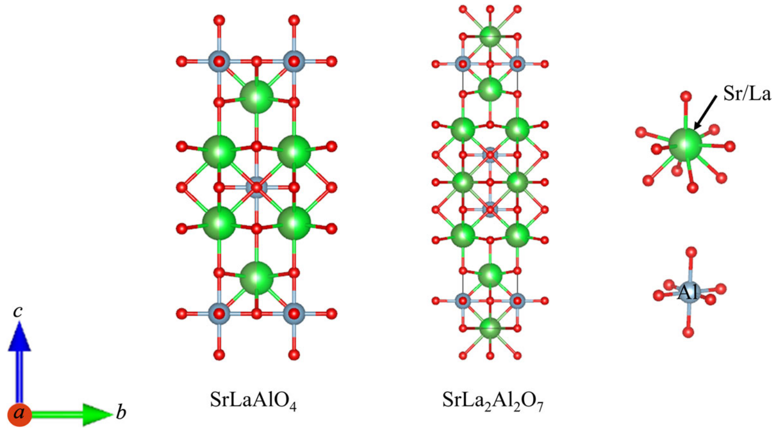

**Fig. 26** Crystal structures of $SrLaAlO_4$ and $SrLa_2Al_2O_7$.

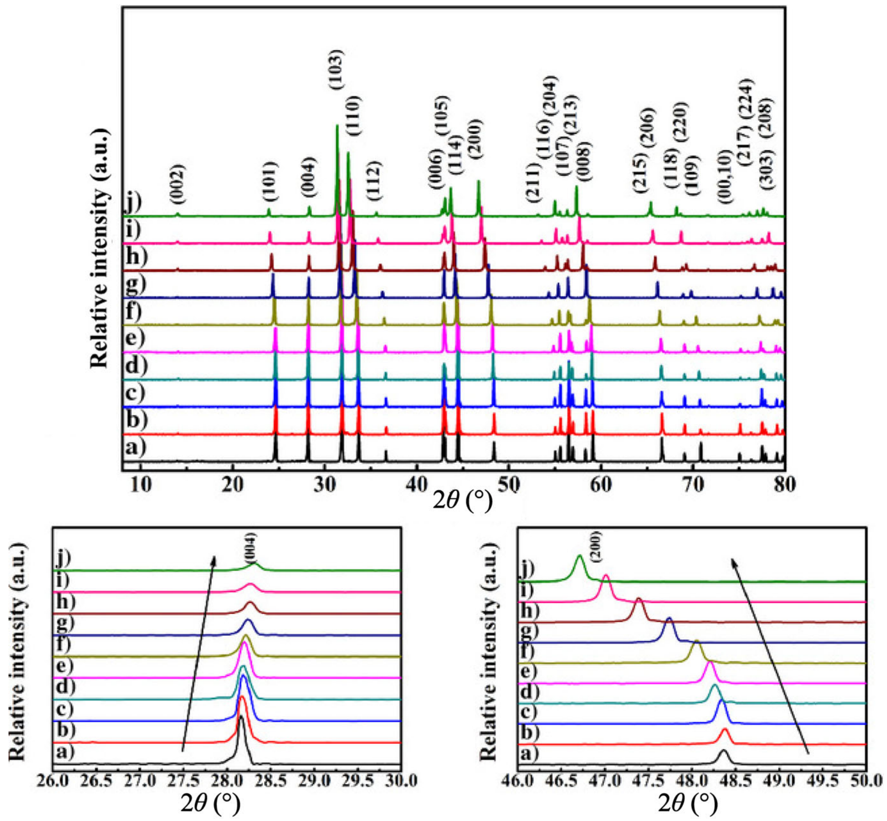

**Fig. 27** XRD powder diffraction patterns of ceramics in (1−$x$)$SrLaAlO_4$–$xSr_2TiO_4$ system with enlarged (004) and (200) diffraction peaks: (a) $x$ = 0, (b) $x$ = 0.025, (c) $x$ = 0.05, (d) $x$ = 0.10, (e) $x$ = 0.15, (f) $x$ = 0.2, (g) $x$ = 0.4, (h) $x$ = 0.6, (i) $x$ = 0.8, and (j) $x$ = 1. Reproduced with permission from Ref. [569], © The American Ceramic Society 2011.

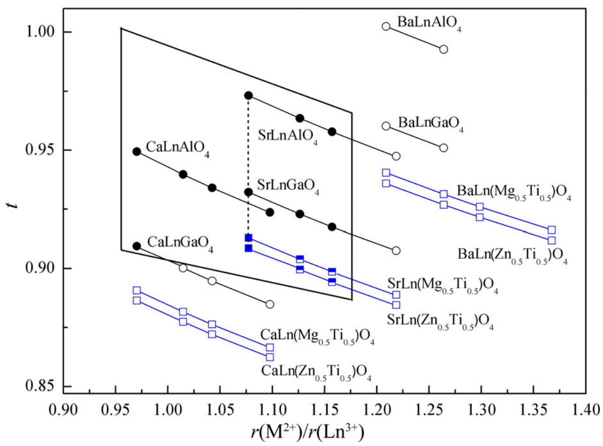

**Fig. 28** Stability of $K_2NiF_4$ structure in $MLnBO_4$ (M = Ca, Sr, Ba; Ln = Y, Sm, Nd, La; B = Al, Ga, ($Mg_{0.5}Ti_{0.5}$), ($Zn_{0.5}Ti_{0.5}$)) compounds in relation to $t$ and $r(M^{2+})/r(Ln^{3+})$. Reproduced with permission from Ref. [573], © The American Ceramic Society 2017.

On the other hand, the R–P structure such as $Sr_{n+1}Ti_nO_{3n+1}$ ($n$ = 1, 2) [576], $SrLn_2Al_2O_7$ (Ln = La, Nd, Sm) [577–581], was also established as $K_2NiF_4$ structure. The interlayer polarization was verified to influence the microstructure and internal stress, and the complete structure information of $SrLn_2Al_2O_7$ ceramics was obtained by TEM. Solid solution of $(Sr_{1-x}Ca_x)_2TiO_4$ [582], $Sr_2Ti_{1-x}Sn_xO_4$ [583], $Sr_2[Ti_{1-x}(Al_{0.5}Nb_{0.5})_x]O_4$ [584], and $(Sr_{1-3x/2}La_x)_2Ti_{1-y}Ce_yO_4$ [585] was prepared to reduce the large $\tau_f$ value of $Sr_2TiO_4$. Moreover, $Sr_2CeO_4$ was obtained by Dai and Zuo [586], and the substitution of $Ti^{4+}$ for $Ce^{4+}$ in $Sr_2CeO_4$ generated a ceramic with excellent properties of $\varepsilon_r$ ≈ 20.7, $Q$×$f$ value ≈ 115,550 GHz, and $\tau_f$ ≈ −1.8 ppm/℃.

## 2.6 Other system and machine learning in MWDCs

Although the pseudo phase diagrams contain various primary systems, some ceramics such as $CeO_2$, $MgAl_2O_4$, $Ca_3Ln_2W_2O_{12}$, and $Ln_2MoO_6$ (Ln = La, Y) do not classify. It is difficult to arrange those ceramics to any phase diagram and the relevant reports are relatively less, and thus, the investigations about the mentioned ceramics are listed in this section. $Ce_{0.75}Y_{0.25}O_{1.875}$ ceramic was indexed as $CeO_2$ phase, and the grain size changed from 0.64 to 1.23 μm contributing to a higher $Q$×$f$ value [587]. The $\tau_f$ value of $(1-x)Bi_2(Li_{0.5}Ta_{1.5})O_7$–$xTiO_2$ was tuned to –1.45 ppm/℃ with $x$ = 0.04 [588]. $0.875CeO_2$–$0.125TiO_2$ composition possessed properties of $\varepsilon_r \approx 27.38$, $Q\times f$ value ≈ 12,950 GHz, and $\tau_f \approx -2.49$ ppm/℃, which could meet the criterion of practical application [589]. $MgAl_2O_4$ transparent ceramic was designed and optimal microwave dielectric properties were obtained: $\varepsilon_r \approx 8.2$, $Q\times f$ value ≈ 110,510 GHz, and $\tau_f \approx -74.1$ ppm/℃ [590]. The $(Mg_{0.5}Ti_{0.4})^{3+}$ for $Al^{3+}$ in $MgAl_2O_4$ could reduce the sintering temperature approximately 200 ℃ due to the less concentration of the Al–O bond [591]. Vibrational spectroscopy and microwave dielectric properties of $Ca_3Ln_2W_2O_{12}$ (Ln = La, Sm) were analyzed by Liu and Song [592], and the $\varepsilon_r$ of those two phases were 18.7 and 19.5. $Ln_2MoO_6$ (Ln = La, Y) ceramics possessed a relative permittivity of 14.1–17.1, and the quality factor was 67,090 GHz for $La_2MoO_6$ and 27,760 GHz for $Y_2MoO_6$, respectively [593].

In the wake of the update of computer science, date-driven approaches including data mining and machine learning have been applied in many disciplines for obtaining the obscure quantitative relationships. For material science, machine learning was used to realize the property prediction, composition optimization, and experimental design [594–600]. Qin *et al.* [601] employed five commonly-used algorithms with 32 intrinsic chemical, structural, and thermodynamic features for modeling to predict low permittivity materials, where a database of 3300 materials has not been reported and the distribution of permittivity in virtual space of materials was shown in Fig. 29. Quantitative prediction of the $Q\times f$ value of gillespite-type $ACuSi_4O_{10}$ (A = Ca, Sr, Ba) ceramics was obtained by machine learning, and the results of $(Ca_xSr_{1-x})CuSi_4O_{10}$ and $(Ba_ySr_{1-y})CuSi_4O_{10}$ ceramics matched well with the experimental $Q\times f$ value, as shown in Fig. 30 [602].

## 3 Conclusions and further outlook

MWDCs with a suitable permittivity, low dielectric loss, and temperature stability are a perpetual pursuit for researchers. Those ceramics offer technoeconomic advantages including integration, lightweight, and reliability. With the continuous exploration, significant progress is presently being made in designing new compounds, analyzing the polarization mechanism

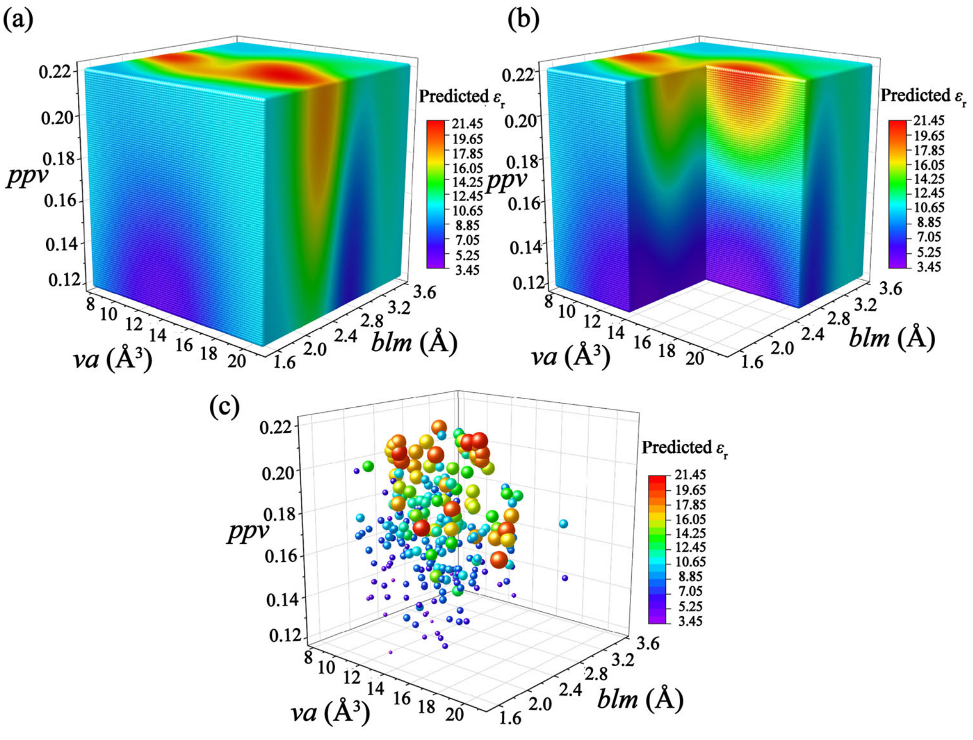

**Fig. 29** Distributions of (a) model predicted relative permittivity in virtual space of materials, (b) cross-section of (a) for a clear view of low permittivity zone, and (c) measured permittivity. The notations of *va*, *blm*, and *ppv* are average cell volume per atom, average bond length, and polarizability per unit volume, respectively. Reproduced with permission from Ref. [601], © The Chinese Ceramic Society 2021.

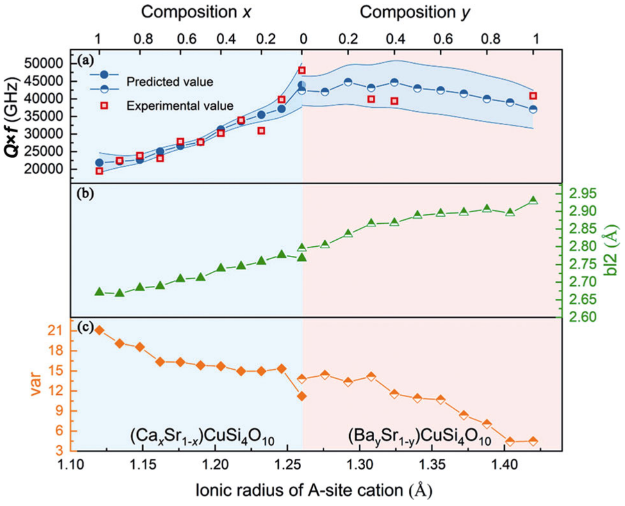

**Fig. 30** Machine learning prediction results. (a) Experimental and predicted $Q×f$ values, (b) feature of bl2 (bond length of A–$O_2$ bond), and (c) feature of var (variance in bond lengths of A–O bond) of $(Ca_xSr_{1-x})CuSi_4O_{10}$ and $(Ba_ySr_{1-y})CuSi_4O_{10}$ ceramics. The shadow area in (a) represents the standard error of 100 trials. Reproduced with permission from Ref. [602], © American Chemical Society 2021.

along with the origin of dielectric loss, and predicting the microwave dielectric properties by theoretical model of machine learning. The relevant computational and experimental methods currently used to probe, predict, and understand intrinsic mechanisms are covered in this review. Because target ceramic system and their associated investigations are so diverse, we provide a brief classification on the composition of ceramics using pseudo phase diagram. The exploration of substitution of the given ceramics or new compounds is listed briefly following the pseudo phase diagram. Experimentally, it appears that substitution and composite ceramics are the most common used methods to optimize the microwave dielectric properties for a given system (reduce dielectric loss or adjust the $\tau_f$ value to near zero). The previous doping researches are concentrated on single ion substitution, while more development of the co-doping (group of two aliovalent cations with a certain mole ratio) appears recently. For the probe of new dielectric materials, the new system usually belongs to germanate and gallate, besides the familiar system of silicate, titanate, niobate, and tantalate. Comparing with conventional solid state reaction method, fabrication techniques containing solution-processed sol–gel method, high energy ball milling method, spark plasma sintering, and microwave sintering have been demonstrated as the promising approaches to improve the properties or sintering behaviors so far. Providing the atmosphere with the volatile element in the sintering procession is a valid method to reduce the pores. Multi-layer ceramic architecture has been verified as a design for temperature-stable ceramics, and the wide application for more system or in the industry is waiting for the exploration.

The influence factor of microwave dielectric properties evolves extrinsic and intrinsic parts. The defects such as porosity, microstructure, and secondary phase are related to the relative density and grain size, which are extrinsic factors. Those results of a unique ceramics can be easily obtained by XRD and SEM, while the investigation of dielectric responded mechanism of intrinsic part is difficult due to the restrain of characterization techniques and the lack of general theory. Theoretically, from Clausius–Mossotti equation, packing fraction, cation valence, distortion of octahedron to the combination of P–V–L theory, lattice dynamics, and THz time-domain spectroscopy with the first-principles calculation, the intrinsic mechanism for MWDCs is gradually created. Recent efforts to employ P–V–L theory and infrared reflectivity spectra to understanding the intrinsic mechanism seem to be an easy and potential approach to draw conclusions for prediction the microwave dielectric properties. However, the development of "try and error" situation in experiments is a long-term procession. Toward this state end, greater fundamental understanding of dielectric response mechanism and increased practical performance metrics are required. The experimental trials and theoretical calculation serve as a database of MWDCs, and then, the machine learning is applied to predict new materials and their microwave dielectric properties. There has been an emerging trend about machine learning to provide new insight to draw a general conclusion to verify the effect of each factor on the variation of microwave dielectric properties. Challenges remain in the reconciliation of conclusion between existing theoretical approaches, the evaluation of P–V–L theory on microwave dielectric properties, and the advancement of first-principles calculation for describing the state of bond. Based on the theoretical analysis of MWDCs and the careful control of extrinsic influence, more comprehensive application-specific analyses to justify their adoption in electronic market may be able to complete.

While there is always a need for fundamental research,

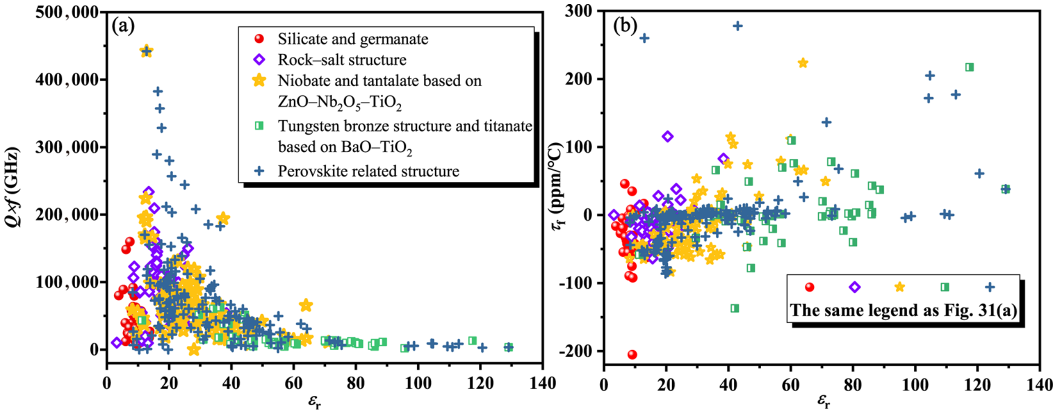

**Fig. 31** Microwave dielectric properties of listed references: (a) $\varepsilon_r$ versus $Q\times f$ values and (b) $\varepsilon_r$ versus $\tau_f$ values.

the acceleration of the commercial application of new materials and property optimized ceramics is another persistent target for researchers. This includes ending the limitation of currently available system and exploration materials with stable and excellent properties for electronic market. For example, alternative materials with satisfied microwave dielectric properties equal to perovskite ceramics are required in the industry. With the development of 5G and 6G, there is an urgent need for ceramics with ultra-low dielectric constant (< 5), low dielectric loss, and excellent temperature-stability in high frequencies. The compounds of borate, aluminate, silicate, and fluoride with low polarization should take into consideration as promising candidate. It may be a direction for discovering composite materials consisted of ceramics and organics. Meanwhile, reducing the sintering temperature of ceramics for meeting the need of LTCC is a highly challenging issue owing to its advantages in fabrication of electronic devices. On the other hand, the repeatability of microwave dielectric properties and the normalized evaluation method should be emphasized. The advancement of preparation method with simplified procedures should be taken into consideration as well. The investigation combining the discussion of the performance of a simulated and fabricated device with the analysis of fundamental mechanism of structure–property relationship should be more popularized to provide an entire and systematical exploration. As a summary, the microwave dielectric properties listed in the references are presented in Figs. 31(a) and 31(b).

Lastly, we hope this brief progress report helps to understand the recent experimental methods and suggests an insight to take a new research direction for MWDCs.

## Acknowledgements

This work is supported by the National Natural Science Foundation of China (Grant No. 51872037).
